# A versatile *Lepidium sativum* bioassay for use in ecotoxicological studies

**DOI:** 10.1038/s41598-025-17215-7

**Published:** 2025-09-23

**Authors:** Viola Maria Schulz, Claudia Scherr, Stephan Baumgartner, Alexander Tournier

**Affiliations:** 1 Institute of Integrative Medicine, University of Witten/Herdecke, Gerhard-Kienle-Weg 4, 58313 Herdecke, Germany; 2 Society for Cancer Research, Hiscia Research Institute, Arlesheim, Switzerland; 3 Institute of Complementary and Integrative Medicine, University of Bern, Bern, Switzerland; 4 Homeopathy Research Institute, London, England

**Keywords:** *Lepidium sativum*, Garden cress, Bioassay, Plant model, Ecotoxicology, Ecotoxins, Environmental pollution, Ecology, Ecosystem ecology, Environmental economics

## Abstract

This study presents a straightforward and efficient bioassay with garden cress (*Lepidium sativum* L.) for ecotoxicological analyses. Cress seedlings grow upright in hanging plastic bags on chromatography paper soaked with the test substance. Scans of the bags easily allow digital measurement of the length of the seedlings after the growth period. Suitability of this test system was examined by treating cress with different concentrations of several heavy metal compounds (cadmium nitrate, copper sulphate, iron sulphate, lead nitrate, manganese chloride, zinc chloride) and sodium chloride. Germination rate, length of shoot and root, total length, and root-to-shoot ratio of the seedlings were measured. Calculated values for half maximal effective concentration (EC50) showed that the used heavy metal compounds affected root length, total length, and root-to-shoot ratio of cress in the following order of toxicity: copper > cadmium > iron > lead > zinc > manganese. The results of our experiments indicate that the cress bioassay presented is an efficient and suitable approach for future investigations on water-soluble ecotoxicologically relevant substances.

**Supplementary Information:**

The online version contains supplementary material available at 10.1038/s41598-025-17215-7.

## Introduction

The observed increase in environmental pollution by ecotoxic substances is mostly attributable to human agricultural and industrial activities^1–3^. Ecotoxins such as heavy metals get into the environment as a result of their use in fertilizers, fungicides, and industrial activities like mining or metallurgic industry^4–6^. Heavy metals can accumulate in soil and in plants, eventually leading to the pollution of groundwater^7^.

Once absorbed by plants used for humans and animals nutrition, they enter the food chain and can have a destructive impact on health^3,8,9^. Furthermore, it has been proposed that the presence of such ecotoxins may contribute to antibiotic resistance^10,11^. As heavy metals are not biodegradable, they have been found to be useful as key markers and reliable indicators of a contaminated environment^12^. In instances where conducting chemical analyses of toxins is not a viable option, the use of plant bioassays is necessary to gain insight into the environmental and ecological risks emanating from contaminated soil and water^13^.

Garden cress (*Lepidium sativum* L., family: *Brassicaceae*) is an interesting organism because it enables a simple and rapid assay to investigate the presence and biological effects of ecotoxins. Cress has been used in various fields of research owing to its modest requirements in terms of growth conditions, high germination rate, short growth period, small size, and uniform growth pattern^14–16^. Bioassays using cress are well suited to examining different environmental parameters such as soil quality^17^water quality^18^(micro)plastic in water^19^metals in sewage sludge^20^ and the influence of ecotoxins such as heavy metals^20,21^. Thanks to its high salt tolerance, cress can also serve as a model organism for moderate halophytes and is used to examine the influence of salt stress^14,22^. The salinisation of soils, often caused by anthropogenic activities, is becoming an increasingly significant problem in agriculture worldwide due to its impact not only on soil and plants but also on aquatic ecosystems^2,23^. Guidelines for the testing of chemicals from organisations such as the Organisation for Economic Co-operation and Development (OECD), the International Organization for Standardization (ISO) or Environmental Protection Agency (EPA) reference bioassays to assess effects on seedling growth as standard methods^24–26^. Cress seedlings are used in bioassays following such guidelines^27–29^.

The objective of the present study was to establish a simple and efficient test system for water-soluble substances using garden cress as a test organism. The intention was to develop a fast and at the same time precise test system, and to allow assessment of germination rate as well as root and shoot development. We performed an overview of bioassays using *Lepidium sativum* described in the scientific literature since the year 2010. Many of these models use comparably time-consuming methods to cultivate and evaluate cress seedlings (involving e.g. manual handling of single seeds) and/or disturb the proper development of the roots (e.g. by horizontal growth in Petri dishes). We therefore refined a bioassay that had previously been developed by our working group^30^. In this bioassay, cress grows on chromatography paper soaked with the test substance in hanging plastic bags, thereby enabling an upright growth of the seedlings. After few days of growth, the bags were scanned, and a computer analysis was used to assess cress shoot and root growth.

In order to assess the suitability and practicability of this bioassay, cress seedlings were treated with different concentrations of the following water-soluble heavy metal compounds: cadmium nitrate, copper sulphate, iron sulphate, lead nitrate, manganese chloride, and zinc chloride. In addition, the seedlings were also grown in different concentrations of sodium chloride as example for a salt promoting the salinisation of soils. The main evaluation parameters were the germination rate of the cress seeds, the length of shoot and root, total seedling length, and root-to-shoot ratio. Our results indicate this cress model to be a valuable addition to the existing ecotoxicological assays.

## Materials and methods

### Literature search

Through a literature search (conducted in September 2020 and updated in September 2024), we aimed to gain an overview of the types of existing bioassays using garden cress as a test organism. The databases PubMed (National Center for Biotechnology Information, Bethesda, USA) and Web of Science (Clarivate Analytics, Philadelphia, PA, USA) were queried to find publications which reported the use of cress models over the years 2010–2024. The search was limited to studies in English. In PubMed search terms were ‘garden cress’ or ‘*Lepidium sativum*’ and in Web of Science ‘*Lepidium sativum*’, in combination with the terms ‘length or growth’ to focus on studies with corresponding outcome. The resulting list of articles was processed using EndNote (version X9, Clarivate Analytics, Philadelphia, USA). Included in the further analysis were publications that described a bioassay with garden cress as test organism, and excluded were studies that focused on other research topics (e.g. examining antidiabetic effects of ingredients of cress). From each of the included studies, we extracted the types of growth containers and procedures used for seedling length measurements, if applicable.

### Description of the bioassay

Garden cress seeds (batch BI15042/19, harvest 2018, Bingenheimer Saatgut AG, Echzell, Germany) were sorted by machines of the supplier company Bingenheimer Saatgut AG. First, a machine with sieves was used to select seeds with sizes 1.50–1.75 mm. The second machine sorted the seeds into five groups depending on their weight; the middle fraction with seeds weighing approximately 2.0–3.5 mg was used for the experiments. Finally, cress seeds were also sorted by hand to discard damaged seeds or seeds with altered phenotypes or traces of mould. The seeds were not sterilised prior to the experiments.

LD-PE plastic pressure lock bags (*microsnap*^®^, flexico, Kunststoff-Schmidt, Essen, Germany) were used. These had two factory-set punch holes in the area above the zipper so they could be hung on rods during the growth period. Half a sheet of chromatography paper (2043 A, Schleicher and Schuell, Dassel, Germany; 140 mm x 170 mm; used in size of 140 mm x 85 mm) was inserted into each bag. Chromatography paper was selected over filter paper due to its lower porosity and more uniform pore structure and surface, which provide better conditions for germination.

Aqueous solutions were prepared using water-soluble heavy metal compounds cadmium nitrate (cadmium nitrate tetrahydrate; Cd(NO_3_)_2_ * 4H_2_O; Merck, Darmstadt, Germany), copper sulphate (copper(II) sulphate pentahydrate; CuSO₄ * 5 H₂O; Fluka, Buchs, Switzerland), iron sulphate (iron(II) sulphate heptahydrate; FeSO₄ * 7 H₂O; Fluka, Buchs, Switzerland), lead nitrate (lead(II) nitrate; Pb(NO_3_)_2_; Merck, Darmstadt, Germany), manganese chloride (manganese(II) chloride dihydrate; MnCl_2_ * 2H_2_O; Merck, Darmstadt, Germany), and zinc chloride (ZnCl_2_; Merck, Darmstadt, Germany) as well as with sodium chloride (NaCl; Fluka, Buchs, Switzerland). The chemicals used had a purity of at least 99%. For each substance, solutions were prepared with the seven concentrations 0.0001 mM, 0.001 mM, 0.01 mM, 0.1 mM, 1 mM, 10 mM, and 100 mM in purified water (reverse osmosis system SEPTRON Line 10 VAL; BWT Aqua AG, Aesch, Switzerland). Cress seedlings grown in purified water served as control. 3 ml of each solution was pipetted into each bag by pipetting lengthwise onto the upper half of the chromatography paper. Three such bags were prepared for each concentration and control. After the paper had absorbed the fluids, 16 cress seeds were placed into each bag. The bags were left open to allow the cress seeds access to air. The bags were then hung upright in a cardboard box (dimensions 40 × 27 × 19 cm) on two bamboo poles (diameter 0.3 cm, length 30 cm), covered from above with a black felt cloth. Hanging from the poles, the bags had no contact with the bottom of the cardboard box. A sheet of cardboard also hung from the poles served as separation between the bags belonging to the different treatment groups. The cardboard box was placed in a polystyrene box (outer dimensions 55 × 36 × 40 cm; wall thickness 3 cm) with a polystyrene lid, which was closed to ensure the cress germinated and grew in darkness. After three hours of soaking at room temperature, the cress seeds developed a transparent layer of slippery mucus. This layer facilitated the manual alignment of the seeds side by side in a horizontal row 9 cm above the bottom of the chromatography paper (video of this process is available in supplement material as Supplementary Video S1). To ensure that the seeds have sufficient access to air, 1 cm space was left between the outermost seeds and the edge of the chromatography paper. After aligning the seedlings, the bags were carefully opened to assure they have not been accidentally closed and hung again on the poles in the cardboard box. The temperature in the cardboard boxes during the growth period was monitored and recorded using temperature chips (Endo Therm GmbH, Arlesheim, Switzerland), which were placed in bags next to the bags with the cress seedlings. The polystyrene boxes were placed in an air-conditioned room at 14.5 °C ± 0.5 °C for 120 h. Experiments involving seven substances at seven concentrations were conducted three times, each on a separate, non-consecutive day, representing three independent experiments.

### Digital processing of images and measurement of seedling length

Following a 120-hour growth period, the bags containing the cress seedlings were scanned (EPSON Perfection V600 with the software ‘Epson Scan’, Seiko Epson Corporation, Suwa, Nagano, Japan). With a weighting plate from above, bags were gently and evenly pressed to the scanner glass. Millimetre paper was scanned alongside each bag to allow calibration in the subsequent analysis. During scanning, all bags were carefully examined for any fungal growth; any such infection was documented and the corresponding bag removed from statistical analysis.

**Fig. 1:**
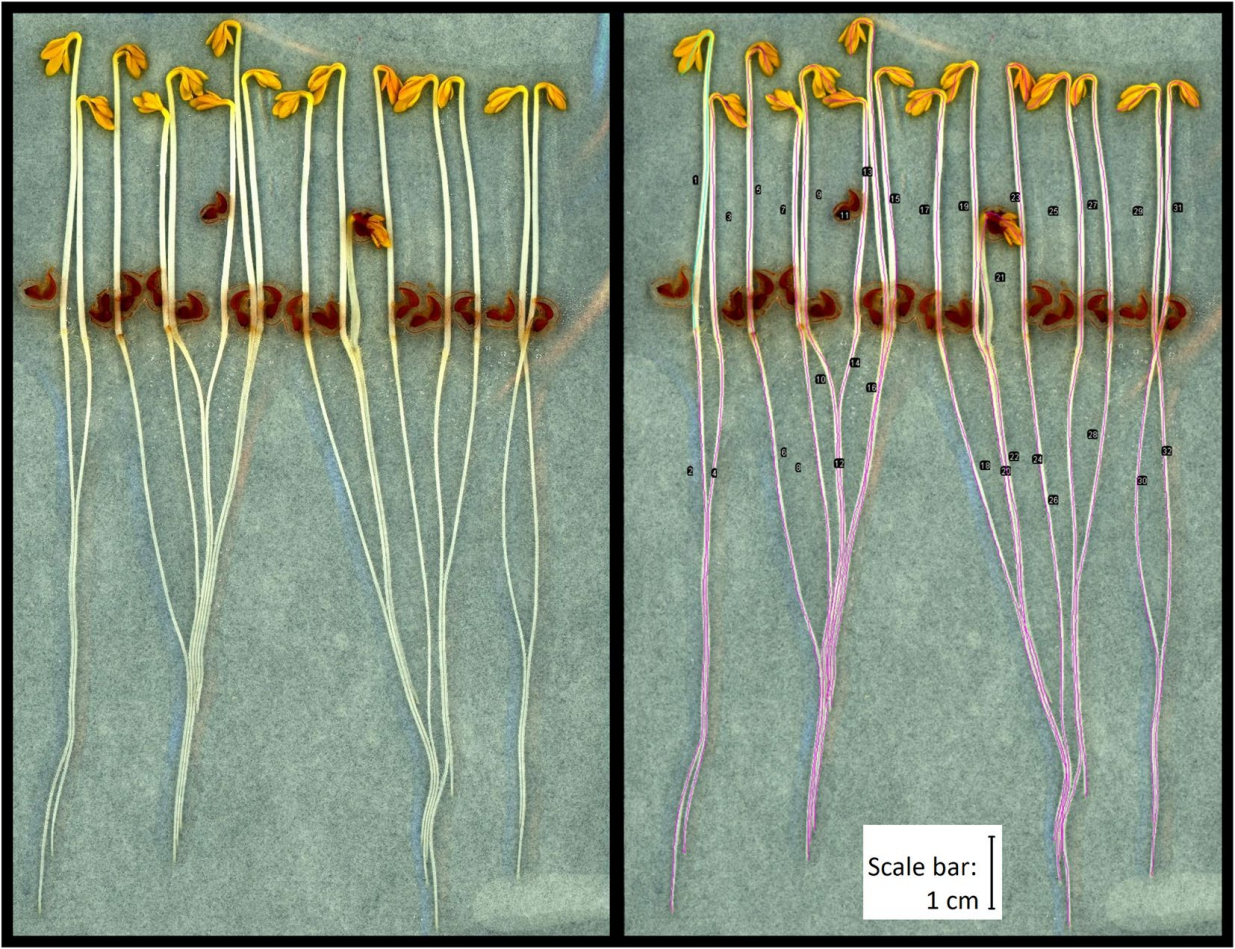
Scan of cress seedlings grown on chromatography paper in LD-PE bag after the growth period of 5 days, without (left) and with (right) ImageJ lines to mark the shape of the seedling. The total length of the seedling is marked in pink. The upper part of the first line is shown in bright blue-green illustrating the differentiation of shoot length from total length. The scale bar represents 1 cm.

The length of the seedlings was measured with the help of a digital tablet (Windows Surface Pro 2017, Microsoft, Redmont, Washington, United States of America) and analysed using the freely available software ImageJ (version 1.52n with Java 1.8.0_112)^31^ with the plugin “Cress Measure Tool” which allows a curve length measurement of the seedlings (provided in Supplementary Plugin S4). This plugin was created based on the ImageJ plugin “Segmented Freehand Line Tool” by Jan Eglinger, which we modified in a way that allows the user to end a drawn line and immediately start a new one by pressing a defined key one the keyboard. For each measurement, the seedling length is measured by manually drawing a line along the length of the seedling on the scan of the bag as it appears on the tablet (see Fig. 1). The plugin allows the user to draw the line separately for shoot and root without lifting the pen. Calibration is performed at the start of each measuring series with the help of the millimetre paper on the scans. We worked with a Wacom pen for tablets (Wacom Bamboo Ink CS-321).

The starting point of each seedling was defined as the tip of the longest yellow leaf. The transition between the shoot and the root is easily identifiable as the root is thinner than the shoot. In addition, the beginning of the root often has a brownish colour, and the root also has little root hairs in contrast to the shoot. Seedlings that did not germinate or have been growing beside the chromatographic paper were not measured but noted. Hidden parts of seedlings were also not measured. Details of the exact measuring procedure, a video demonstrating the method, the ImageJ plugin, and an Excel template file example are available as online supplementary material (as Supplementary Method S2, Supplementary Video S3, Supplementary Plugin S4, Supplementary Excel File S5). The outcome parameters were shoot length, root length, total length, and root-to-shoot ratio. The germination rate was calculated as a percentage of germinated cress seeds of the total amount of seeds tested. A seed was considered germinated when the tip of the root became evident, protruding from the seed coating. Seedlings that grew beside the chromatographic paper were considered for the calculation of the germination rate.

In order to estimate the measurement error (due to the manual seedling tracing procedure on the tablet), 48 cress seedlings (grown in purified water in three bags) were measured three times each. The three measurements’ mean value and standard deviation were determined for each seedling. Afterwards, the overall mean, the overall standard deviation and the coefficient of variation (CV, calculated as ratio of standard deviation and mean) were calculated for each parameter. Analysed parameters were shoot length, root length, total length, and root-to-shoot ratio.

### Statistical evaluation

Statistica (Version 13.3 for Windows, TIBCO Software Inc., USA) was used for all statistical analyses. Analyses of variance (ANOVA) were performed for root length, shoot length, total length, and root-to-shoot ratio with the independent parameters experimental number (*n* = 3), substance (*n* = 7), and concentration (*n* = 7).

Furthermore, for each substance, the half maximal effective concentration (EC50) was calculated for shoot length, root length, total length and root-to-shoot ratio of the seedlings. EC50 is defined as the effective concentration causing 50% of the effect for a given evaluation parameter. For the determination of EC50, we used the Hill equation using non-linear regression. Although other methods, such as the probit model, exist, we employed the Hill equation for several reasons, which are discussed elsewhere^32^. The EC50 was calculated with the formula

$$\:f\left(x\right)=Max-\frac{Max}{1+({\frac{x}{{EC}_{50}})}^{Hill}}$$

with the variables f(x) = evaluation parameter, Max = maximum value of the parameter, $\:x$ = concentration in mM and $\:Hill$ = Hill slope.

The coefficients of variation of the EC50 values were calculated as the ratio of standard error and EC50 value (as percentage).

Non-germinated seeds were included only in the calculation of the germination rate, while they were excluded from all other statistical analyses, including length parameters and root-to-shoot ratio.

## Results

### Literature search

The literature search in PubMed comprising the timeframe of ten years (2010 to 2020) with the search terms ‘garden cress’ or ‘lepidium sativum’ yielded 350 publications. Out of these, 127 publications were excluded as they did not describe bioassays with garden cress. 223 publications that used cress as an experimental test organism were included for further analysis. The literature search in Web of Science yielded 279 results; 258 of these described bioassays with garden cress (21 publications were excluded, unrelated topics). In total, after removing duplicates of the two databases, 381 publications were included and analysed regarding bioassays with garden cress as test organism.

In this literature search, we found five publications that used the bioassay with hanging plastic bags to cultivate cress seedlings. Three of them were published by our working group^30,33,34^ and two were published by another working group following the method of Baumgartner et al.^35,36^.

In other studies, cress was grown in various types of containers. Most often different kinds of Petri dishes^37,38^, pots^39^ or test plates^40^ were used. In addition, for some investigations, the cress was grown in bioreactors^41^, glass jars^42^, trays^43^, glass vials^44^, and different other kinds of containers^45–47^. In some studies, the seedlings were grown in fields^48^.

Different methods were described to measure the seedling length. In some studies, the measurement was performed manually, for example using a ruler^49^, meter scale^50^, measuring tape^29^, caliper^51^ or digital gauge^27^. Sometimes, a microscope was used^49^. With the software ImageJ, cress seedlings on digital images could be measured digitally^39,52^. Furthermore, besides ImageJ, other software programs were also used in some studies like Image Tool 3.0 for Windows (UTHSCSA, San Antonio, USA) which was often combined with the phytotoxicity test kit “Phytotoxkit microbiotest”^42,53^. The publications of our working group described a measurement method by a computer-aided curve length measurement using a graphics tablet^30,33,34^. We modernised the hardware and software of this approach for the present study.

In September 2024, an update of the literature search was performed in both databases (with the same search terms as in the previous search) which yielded additional 226 publications in PubMed with 81 articles about bioassays with cress. In Web of Science, 190 new publications were found and 153 of these were about cress used as test plant. Overall, we identified only three substantially different bioassays with cress through this updated search. A small-scale terrestrial model ecosystem (STME) mimicking a natural ecosystem was described in one publication^54^. Another group presented a micropot made with 3D printing technology to examine plant growth under microgravity conditions^55^. No study was found in the updated search that has used the bioassay with hanging plastic bags we present in this publication.

Overall, 559 articles were identified that described bioassays with cress as a test organism. We analysed 533 of these articles in terms of the characteristics of the cress model used (full text of 26 publications could not be found). A list of the 533 analysed bioassays with cress is provided in the supplement material (Supplementary List S6).

### Measurement error and natural variability

The measurement error due to the tablet measurement procedure was determined by three times measuring 48 seedlings grown in purified water. Coefficients of variation were between 0.39 and 1.83% (see Table 1).

**Table 1:** Measurement error due to the seedling length measurement procedure using a digital tablet and imagej. Cress seedlings (*n* = 48) in three different bags cultivated in purified water were measured three times each. The overall mean value of the measurements, the overall standard deviation (SD) and the coefficient of variation (CV) were calculated for each parameter. Analysed parameters were shoot length, root length, total length, and root-to-shoot ratio.

Parameter	*n*	Measurements	Mean	SD	CV
Shoot length [cm]	48	3	3.95	0.05	1.19%
Root length [cm]	48	3	6.34	0.05	0.76%
Total length [cm]	48	3	10.30	0.04	0.39%
Root/shoot ratio	48	3	1.61	0.03	1.83%

For a comparison of the pure measurement error with the natural variability of the growth of cress regarding the four evaluation parameters, ten bags with overall 154 cress seedlings grown in purified water were analysed to determine the natural variability, estimated by the corresponding coefficients of variation, which were between 11.01 and 24.95% (see Table 2).

**Table 2:** Natural variability of length of Cress seedlings (*n* = 154) grown in purified water. Mean value, standard deviation (SD), and coefficient of variation (CV) were calculated for each parameter (shoot length, root length, total length, and root-to-shoot ratio).

Parameter	*n*	Measurements	Mean	SD	CV
Shoot length [cm]	154	1	3.90	0.43	11.01%
Root length [cm]	154	1	6.10	1.52	24.95%
Total length [cm]	154	1	10.00	1.81	18.05%
Root/shoot ratio	154	1	1.55	0.36	22.91%

### Experiments with ecotoxic substances

**Fig. 2:**
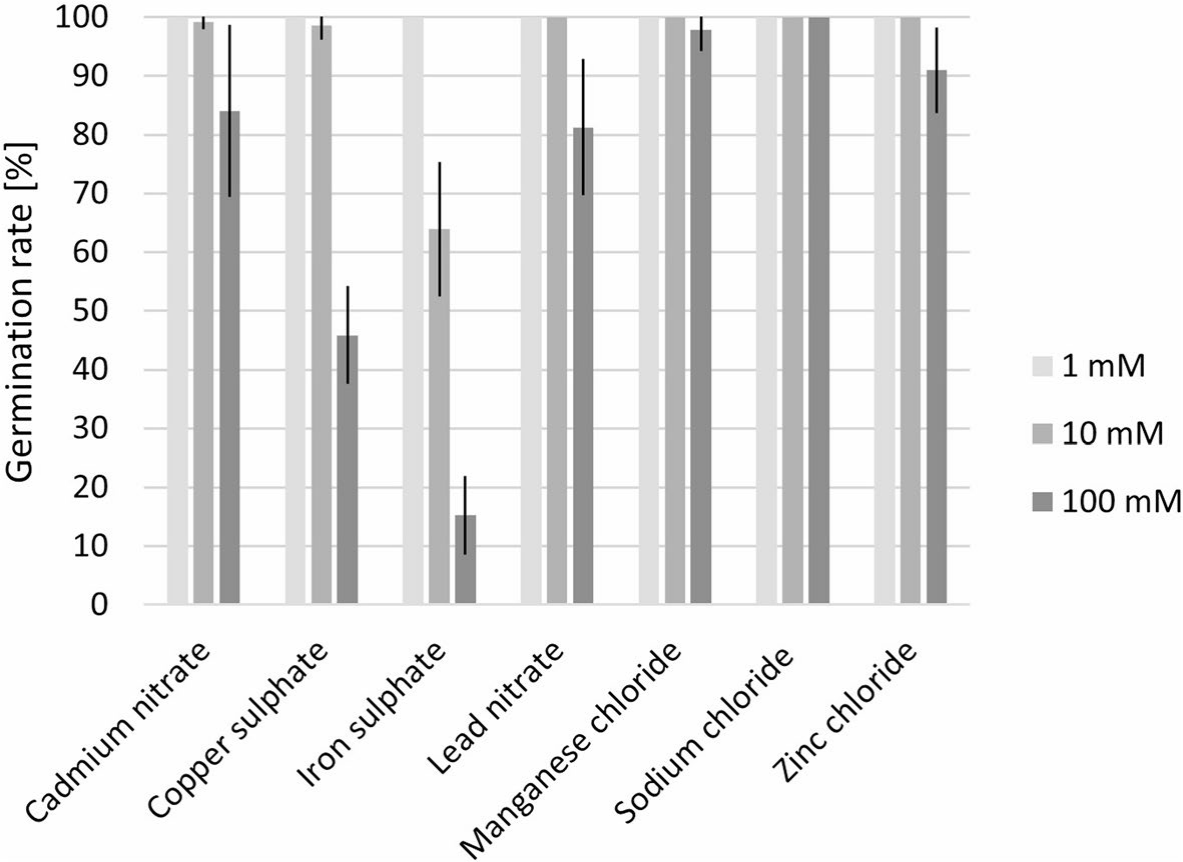
Germination rate of cress seeds treated with ecotoxic substances (cadmium nitrate, copper sulphate, iron sulphate, lead nitrate, manganese chloride, sodium chloride, and zinc chloride) in different concentrations. The germination rate in purified water was 100%. Only the effects of the concentrations 1 mM, 10 mM, and 100 mM are shown in this figure as lower concentrations did not affect the germination rate. Error bars represent the standard deviation.

The germination rate of the cress seeds was reduced by treatment with iron sulphate starting at a concentration of 10 mM, and with cadmium nitrate, copper sulphate, lead nitrate, and zinc chloride at 100 mM (Fig. 2). Neither manganese nor sodium chloride influenced the germination rate at the concentrations used in a relevant way.

**Fig. 3:**
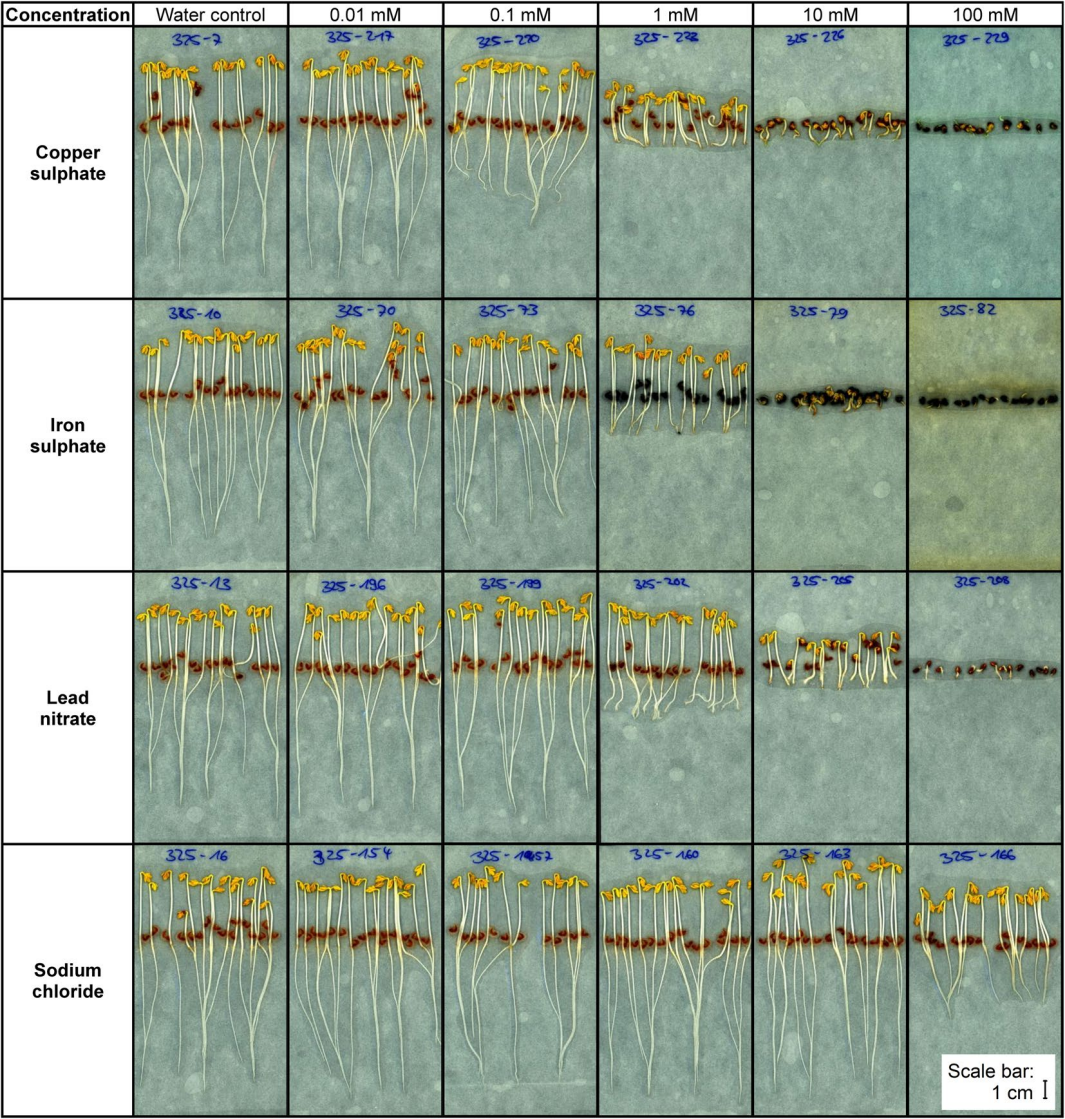
Representative examples of scans of cress seedlings treated with copper sulphate, iron sulphate, lead nitrate, sodium chloride, and purified water as control. Only scans of seedlings treated with concentrations between 0.01 mM to 100 mM are shown in this figure, as no evident changes in seedling growth in solutions with lower concentrations were observed. The scale bar represents 1 cm.

Phenotypic changes and impaired growth of the seedlings were visible to the human eye after treatment with a concentration as low as 0.1 mM cadmium nitrate, copper sulphate, and iron sulphate, with 1 mM lead nitrate, manganese chloride, and zinc chloride, and with 100 mM sodium chloride. Representative examples of scans of cress seedlings treated with copper sulphate, iron sulphate, lead nitrate, sodium chloride, and purified water as control are shown in Fig. 3 (example scans of all used substances are shown in the supplement in Supplementary Figure S7). At higher concentrations, some substances like lead nitrate led to the development of more root hairs, while other substances like iron sulphate affected the colour of seed coats and root tips.

**Fig. 4:**
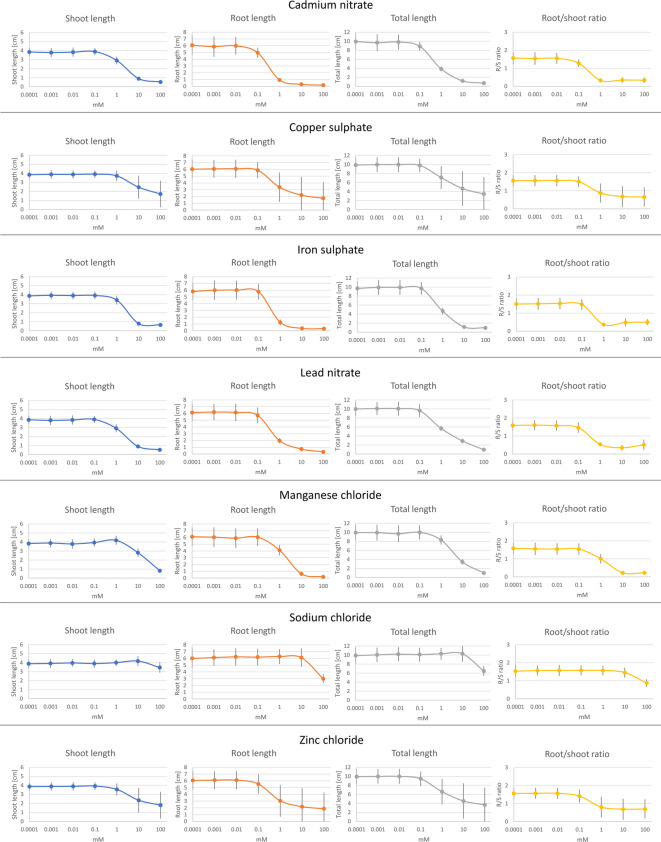
Shoot length, root length, and total length as well as root-to-shoot ratio of cress seedlings treated with cadmium nitrate, copper sulphate, iron sulphate, lead nitrate, manganese chloride, sodium chloride, and zinc chloride at 0.0001 mM, 0.001 mM, 0.01 mM, 0.1 mM, 1 mM, 10 mM, and 100 mM concentrations. Error bars represent the standard deviation.

The measured growth parameters of cress seedlings treated with seven different ecotoxic substances and purified water as control is shown in Fig. 4. The shoot length was reduced at increasing concentrations starting at 1 mM of cadmium nitrate, iron sulphate, lead nitrate, and zinc chloride as well as at 10 mM of copper sulphate and manganese chloride. Root length and total length decreased starting at 0.1 mM concentrations of cadmium nitrate and at 1 mM of copper sulphate, iron sulphate, lead nitrate, manganese chloride, and zinc chloride. The root-to-shoot ratio was reduced after treatment of the cress with cadmium nitrate at a concentration of 0.1 mM and 1 mM of copper sulphate, iron sulphate, lead nitrate, manganese chloride, and zinc chloride. Sodium chloride caused a decrease of shoot length, root length, total length, and root-to-shoot ratio at 100 mM.

The results of the two-way ANOVA for the evaluation parameters shoot length, root length, total length, and root-to-shoot ratio of cress seedlings treated with seven substances in seven concentrations in three independent experiments are shown in Table 3. Comparison of the F-values shows that the influence of the concentration of the different substances was much larger than the influence of the three independent experiments or their interaction.

**Table 3:** Results of two-way ANOVA performed for root length, shoot length, total length, and root-to-shoot ratio of Cress seedlings with the independent parameters experiment number (*n* = 3), substance (*n* = 7), and concentration (*n* = 7, varying between 0.0001 mM and 100 mM). Significant values are printed in bold.

Substance	Statistical parameter	Shoot length		Root length		Total length		Root/shoot ratio	
		F Ratio	*p* Value	F Ratio	*p* Value	F Ratio	*p* Value	F Ratio	*p* Value
Cadmium nitrate	Experiment number	**5.15**	**0.01**	0.63	0.53	1.84	0.16	**4.60**	**0.01**
	Concentration	**1780.11**	**< 0.01**	**1165.54**	**< 0.01**	**1577.03**	**< 0.01**	**906.53**	**< 0.01**
	Interaction	1.40	0.16	**1.87**	**0.03**	**1.82**	**0.04**	**3.36**	**< 0.01**
Copper sulphate	Experiment number	**27.80**	**< 0.01**	**6.71**	**< 0.01**	**12.60**	**< 0.01**	1.81	0.16
	Concentration	**1051.70**	**< 0.01**	**1053.31**	**< 0.01**	**1131.85**	**< 0.01**	**1006.99**	**< 0.01**
	Interaction	1.20	0.29	0.25	1.00	0.18	1.00	**1.94**	**0.03**
Iron sulphate	Experiment number	**8.60**	**< 0.01**	1.03	0.36	0.15	0.86	**9.15**	**< 0.01**
	Concentration	**886.33**	**< 0.01**	**620.19**	**< 0.01**	**789.38**	**< 0.01**	**501.72**	**< 0.01**
	Interaction	1.47	0.13	0.92	0.53	0.72	0.73	**2.64**	**< 0.01**
Lead nitrate	Experiment number	**4.77**	**0.01**	0.18	0.84	0.84	0.43	0.90	0.41
	Concentration	**1422.19**	**< 0.01**	**1101.53**	**< 0.01**	**1416.68**	**< 0.01**	**793.69**	**< 0.01**
	Interaction	**1.83**	**0.04**	1.27	0.23	1.27	0.23	**3.59**	**< 0.01**
Manganese chloride	Experiment number	**11.34**	**< 0.01**	**6.05**	**< 0.01**	**8.54**	**< 0.01**	2.31	0.10
	Concentration	**1009.20**	**< 0.01**	**838.75**	**< 0.01**	**1045.49**	**< 0.01**	**773.75**	**< 0.01**
	Interaction	**1.96**	**0.03**	0.87	0.58	1.02	0.43	1.19	0.29
Sodium chloride	Experiment number	**17.23**	**< 0.01**	**3.55**	**0.03**	**5.76**	**< 0.01**	**5.15**	**0.01**
	Concentration	**30.36**	**< 0.01**	**139.48**	**< 0.01**	**119.14**	**< 0.01**	**120.15**	**< 0.01**
	Interaction	0.80	0.65	1.07	0.38	0.84	0.61	1.64	0.08
Zinc chloride	Experiment number	**23.00**	**< 0.01**	**4.21**	**0.01**	**8.79**	**< 0.01**	2.71	0.07
	Concentration	**938.30**	**< 0.01**	**956.72**	**< 0.01**	**1025.62**	**< 0.01**	**910.30**	**< 0.01**
	Interaction	1.40	0.16	0.31	0.99	0.27	0.99	**1.76**	**0.05**

Table 4 presents the calculated EC50 values for all the substances tested (except sodium chloride due to too small effects in the concentration range chosen, see Figs. 3 and 4) of the four parameters, along with 95% confidence interval and the coefficient of variation.

**Table 4:** Half maximal effective concentration EC50 [mM] of heavy metal compounds cadmium nitrate, copper sulphate, iron sulphate, lead nitrate, manganese chloride, and zinc chloride regarding the parameters shoot length, root length, total length, and root-to-shoot (R/S) ratio of Cress seedlings. Values in brackets display the 95% confidence interval (CI). Values for variation coefficient (CV) are expressed in %. EC50 values for sodium chloride could not be calculated (n.a.) due to the small effects in the concentration range chosen.

EC50 [mM] 95% CI CV	Shoot length	Root length	Total length	*R*/S ratio
Cadmium nitrate	3.23 [2.97–3.48] 4.03%	0.31 [0.27–0.34] 5.45%	0.70 [0.64–0.77] 4.66%	0.37 [0.32–0.43] 7.75%
Copper sulphate	3.27 [3.00–3.53] 4.11%	0.13 [0.12–0.15] 5.09%	0.43 [0.39–0.48] 5.51%	0.16 [0.13–0.18] 8.52%
Iron sulphate	3.85 [3.54–4.15] 4.00%	0.53 [0.44–0.63] 9.05%	0.95 [0.87–1.02] 3.99%	0.52 [0.44–0.6] 7.6%
Lead nitrate	13.65 [12.72–14.58] 3.46%	0.58 [0.52–0.64] 5.26%	1.95 [1.74–2.16] 5.5%	0.78 [0.62–0.95] 10.72%
Manganese chloride	26.28 [24.2–28.36] 4.04%	1.82 [1.63–2.02] 5.51%	5.4 [4.88–5.93] 4.98%	1.82 [1.6–2.05] 6.22%
Zinc chloride	11.71 [10.85–12.57] 3.75%	1.07 [0.96–1.17] 4.82%	2.90 [2.61–3.19] 5.02%	1.29 [1.11–1.47] 7.13%
Sodium chloride	n.a.	n.a.	n.a.	n.a.

## Discussion

### Literature search

533 articles describing bioassays with cress as test organism have been found through the literature search in the timeframe of the years 2010–2024. Our search strategy was restricted to PubMed and Web of Science and could have overlooked some publications only available in other databases. However, we assume that a well-established bioassay would have appeared in studies published in these two widely used databases. The extracted information from the articles found about plant models using cress provided a good overview of methodological approaches. We were inspired by some publications which used ImageJ to measure the seedling length.

Our literature search yielded only five studies that applied a bioassay with hanging plastic bags as containers to grow cress seedlings^30,33–36^. These were all based on the method described first by Baumgartner *et al*. 2012. No publication was found that described a true curve length measurement method with a digital tablet in combination with an image analysis software. We therefore conclude that to the best of our knowledge the present bioassay has not been described before by another working group.

### Procedure of the bioassay

For our study, we decided to modernise the bioassay based on cress seedlings growing upright in hanging plastic bags. This plant model was already used for previous studies of our and another working group^30,33–36,56^.

The present assay has several advantages. The plastic bags can be packed easily with chromatography paper, and larger amounts can be prepared and stored in a space-saving manner before actually performing experiments. This also allows setting up larger experiments with several hundred bags (corresponding to several thousand seedlings) in a very short time (see below). The plastic bags with the seeds are vertically suspended on rods what allows dense packaging of seedlings and in turn minimizes space requirements. This allows using smaller growth cabinets and also helps minimizing spatial gradients (e.g. in temperature) what in turn helps minimizing drifts and systematic errors. As an example, 4800 seedlings can be cultivated in 300 bags which can be packed in a volume of 57 × 33 cm.

The hanging plastic bags allow upright seedling growth. Therefore, the different parts of the plants, like shoot and root, are easily visible and distinguishable from each other, enabling a precise measurement of individual seedling length. Besides germination rate, cress seedling length is a parameter often analysed^57,58^. The upright position of the seedlings in the bags offers the opportunity to measure not only the total length of the seedlings but also the root and shoot lengths separately. As an additional analysis parameter, the root-to-shoot ratio can thus be determined. We see advantages in this methodical approach in contrast to bioassays using other containers (such as Petri dishes, pots etc.) in which cress seedlings often grow very close to each other on a plane or in the medium. This makes the measurement of the root length of the seedlings more difficult or even impossible. However, since the root of cress is highly sensitive to different substances it is advantageous that this parameter can be measured accurately^59^. Other working groups implemented gravitropic/upright-oriented seedling growth by using vertical plates, or by facing the dishes down at an angle of approximately 60°^38,60^. The hanging bag method allows the bags to be easily scanned to obtain digital images of the cress plants, which can then be digitally processed to measure seedling length.

Moreover, it is possible to remove the cress seedlings from the bags after the growth period for further chemical analysis^34^.

A potential disadvantage of the hanging bag method is that even if the pressure lock is left open, air exchange in the bag remains limited, which might promote the growth of fungi or bacteria. Because of that, in our experimental set-up, the bags were carefully opened after aligning the seedlings to allow as much air access as possible. If the cress seedlings show unexplainable unusual growth behaviour, an infection with fungi or bacteria could be a reason for it. Fungal infections can lead to a reduced germination rate and also to a decreased growth of the cress seedlings^16,61^. Due to unfavourable conditions in the growth lab or spores on materials like cress seeds, chromatography paper or in the water, the risk of a bacterial infection and fungal development in the bags rises. Nevertheless, our experiences show that fungal or bacterial infections remain very rare with our approach, also due to temperature control in the growth lab (14.5 ± 0.5 °C). In cases of infection, affected bags can be easily identified during scanning and removed from statistical analysis.

A possible further disadvantage of the present bioassay could result from adsorption of certain compounds to the chromatography paper used which would lead to an underestimation of the biological impacts of such compounds. Another possible disadvantage of the present bioassay is that testing of volatile compounds may result in cross-contamination due to evaporation through the opened bags. Furthermore, in case of toxic volatile compounds, corresponding safety measures have to taken to protect the experimenter.

This bioassay provides a laboratory model to test the ecotoxicological impact of water-soluble substances. However, the reaction of cress in our test system could be different in comparison to cress growing in nature under natural conditions as here cress is grown in darkness, at a stable temperature and without soil. The sensitivity of cress to certain substances could be lower in soils with more organic matter^62^. However, varying environmental factors such as temperature and light can also be additional stressors for the plants^63^. A laboratory model, as the present bioassay, allows more stability regarding these external factors.

We found this modernised cress bioassay to be a relatively inexpensive and efficient method for testing ecotoxins. For an experimental series as in our study with 500 bags and each bag filled with 16 cress seeds (amounting to 8000 cress seedlings in total), the total working time was about 27 h (∼3.30 min/bag). 11 h of working time were needed to prepare and perform the experimental part (∼1.30 min/bag), and 16 h were needed for measuring the seedling length with the digital tablet and the ImageJ macro (∼2 min/bag). We thus conclude that the present bioassay is an efficient plant model to test effects of water-soluble substances.

### Computer-assisted parameter measurements

Phytotoxic substances can harm plants, resulting in different signs of damage in terms of germination, growth, and phenotype. Seed germination and root elongation assays were found to be valid for the assessment of phytotoxicity in plant models and for experiments with cress^64,65^. For many bioassays with cress, germination rate and length of the cress seedlings are considered the most relevant criteria in determining the level of toxicity of a given substance^27,49,66^. Accordingly, we decided to use the following five evaluation parameters: germination rate, shoot length, root length, total length, and root-to-shoot ratio. For the reliable analysis of these parameters, obtaining high-quality images of the bags with the seedlings was crucial. We found scanning of the plastic bags to be a fast and reliable method.

Some non-digital manual methods for measuring the length of the cress seedlings (e.g. with a ruler) can be time-consuming, imprecise and often not well suited to measuring curved seedlings. We tried different software such as Root detection (http://www.labutils.de/) or ImageJ plugins like Smart Root^67^ or NeuronJ^68^ that might be suitable for an automatic digital analysis of the seedling length. According to our experiences, none of the software programs tested was reliable in detecting the length of the roots, especially not when roots crossed over each other or grew close to each other. The accurate distinction of shoot and root was also problematic and was not achieved satisfactorily by the software we tested. At the time of writing, the process of correcting the mistakes made by automatic digital tracking algorithms is still more time-consuming than simply tracking each seedling by hand.

Thus, for our study, we performed digital curve length measurements using a digital tablet and ImageJ with the plugin “Cress Measure Tool” (provided in Supplementary Plugin S4). This procedure allows an efficient, reliable and exact measurement of the curved seedling length with distinction of shoot and root and an easy identification and correction of possible mistakes. The pure measurement error was relatively low (variation coefficients of 0.4–1.8% for the four outcome parameters). Compared to the natural variability introduced by the inhomogeneities of the cress seedlings (11–25%), the measurement error is negligible. With the method used, inaccurate measurements e.g. by mistracking can be identified and corrected rapidly. Another advantage of this system is that the specific transition point between shoot and root can be easily specified without lifting the pen, thus avoiding inaccuracies in root vs. shoot identification. New technologies using artificial intelligence will no doubt offer new possibilities in the future, however, our method currently allows fast and reliable measurements of the seedling length.

### Ecotoxicology

In our investigations, cress was treated with the heavy metal compounds cadmium nitrate, copper sulphate, iron sulphate, lead nitrate, manganese chloride, and zinc chloride, as well as with sodium chloride. We chose these substances due to their water solubility and possible harmful influence if accumulated in plants^7,8,21^.

Our results are in agreement with the findings of other studies that the germination and growth of cress are reduced with increasing concentrations of heavy metals^69,70^. The growth of cress seems to be a more sensitive outcome parameter than the germination rate since it is affected at lower concentrations of heavy metal compounds. This hints at a higher resistance of cress seeds against toxins compared to cress seedlings^70^.

Sodium chloride did not affect seed germination at the concentrations we used (which were the same for all compounds). Phenotype and growth of the cress seedlings were less affected by sodium chloride in comparison to the other applied substances at same concentrations. This corresponds to previous studies which show that garden cress is a moderately salt tolerant plant^14,22,71^. A growth reducing effect of concentrations of sodium chloride higher than 100 mM was also observed on cress seedlings in previous studies^14,22^.

With our test system, it was possible to calculate the EC50 of the different ecotoxins for the parameters: shoot length, root length, total length, and root-to-shoot ratio of cress seedlings. The roots of the cress seedlings were affected at lower concentrations of the applied substances in comparison to the shoots. According to these results, the shoots could be less sensitive to the substances than the roots, or the roots accumulate more of the substances than the shoots. For example, according to Gill et al. cadmium accumulation is higher in roots than in leaves of cress seedlings^72^. Diffusion barriers as a control mechanism for nutrient uptake in the roots of the cress seedlings could be a possible explanation for this phenomenon^73^.

The EC50 values indicated that the used heavy metal compounds affected root length, total length as well as root-to-shoot ratio of cress in the following order of toxicity: copper > cadmium > iron > lead > zinc > manganese. This order was also observed in a former study using different compounds of these heavy metals, except iron^74^. However, other studies might arrive at different conclusions regarding this order as the toxicity of metals is also influenced by factors like kind and quality of substrate, length of growth period etc^20^. The order of toxicity of substances depends also on the evaluation parameter: for the influence on the shoot length of the cress seedlings, the EC50 values of our study indicated the order cadmium > copper > iron > zinc > lead > manganese. The variation coefficient of the EC50 values for the four growth parameters was quite low for a plant bioassay, with all values lower than 11%.

In addition, to perform investigations with water-soluble ecotoxins, the present test system can also be interesting for water monitoring and leachate ecotoxicity assessment. It cannot be used for investigations on solid substrates as soil itself. However, a soil extraction method^8,13,21^ can be a possible approach to investigate ecotoxicity of soils with this bioassay. Moreover, this plant model could also provide the possibility to investigate detoxifying substances by adding these to the growth solutions with the toxins. For example, the effects of humic and fulvic acid could be investigated which might be able to reduce the uptake of toxic substances of cress seedlings^75^.

## Conclusion and outlook

We present a straightforward and efficient test system using garden cress as test organism for use in ecotoxicology. This plant model uses hanging plastic bags to enable upright growth of cress seedlings, which allows a precise identification and analysis of the seedlings. With this bioassay, it is possible to measure the length of the seedlings on scans of the bags after the growth period and to distinguish between shoot and root length as evaluation parameters. The use of a digital tablet and the software ImageJ allow a fast, accurate, and reliable measurement of the seedling length. This plant model with cress is fast, simple, and cost effective and introduces a promising option for investigating water-soluble substances such as ecotoxins.

## Supplementary Information

Below is the link to the electronic supplementary material.

Supplementary File 1

Supplementary Video 1

Legend Supplementary Video 1

Supplementary material 2

Supplementary Video 3

Legend Supplementary Video 3

Supplementary File 4

Supplementary Material 5

Legend for Supplementary Material 5

Supplementary Material 6

Supplementary Material 7

## Data Availability

The data supporting the findings of this study can be obtained from the corresponding author upon request.
