# Supplementary material for: A versatile Lepidium sativum bioassay for use in ecotoxicological studies
Source: Sci Rep. 2025 Sep 23;15:32653. doi: 10.1038/s41598-025-17215-7 (PMC12457589; doi:10.1038/s41598-025-17215-7)
Supplement: Supplementary file 1 [file 41598_2025_17215_MOESM1_ESM.pdf]

Journal "Scientific Reports"

A versatile *Lepidium sativum* bioassay for use in ecotoxicological studies

Viola Maria Schulz, Claudia Scherr, Stephan Baumgartner and Alexander Tournier

Address correspondence to: Viola Schulz, MSc, Institute of Integrative Medicine, University of Witten/Herdecke, Gerhard-Kienle-Weg 4, 58313 Witten, Germany.

E-mail: Viola.Schulz@uni-wh.de

Description of supplementary material

Supplementary Video S1: Video of aligning the cress seeds

This video shows how to easily arrange the cress seeds in a row after they have formed a mucus layer and before the growth period.

Supplementary Method S2: Manual for the measurement procedure of seedling length with tablet and ImageJ

Step by step explanation how to measure the length of the cress seedlings with the Windows surface tablet and the software ImageJ with an associated plugin.

Supplementary Video S3: Video of the length measurement procedure with the digital tablet and ImageJ

In this video, the measurement of the scan of one bag with cress seedlings is shown as example how to measure the seedling length with the Windows surface tablet and the software ImageJ with an associated plugin.

Supplementary File S4: ImageJ Plugin “Cress Measure Tool”

This document contains the ImageJ plugin that allows a curve length measurement of the seedlings. The plugin also enables to draw lines separately for shoot and root without lifting the pen but by pressing a defined key on the keyboard where the shoot ends, and the root starts. This plugin was created based on the ImageJ plugin "Segmented Freehand Line Tool" by Jan Eglinger.

Supplementary Excel File S5: Excel template

This Excel file can serve as template for insertion of the data of ImageJ measurements of cress seedlings so that the outcome parameters shoot length, root length, total length, and root-to-shoot ratio can easily be calculated.

Supplementary List S6: List of bioassays with garden cress extracted from publications found in PubMed and Web of Science (years 2010 - 2024)

This list provides an overview on 533 publications reporting on bioassays with cress identified in a literature search in the databases PubMed and Web of Science (years 2010 to 2024). In this list, containers used to grow the cress, the measurement method (if seedling length measurement was an outcome parameter), and stated references regarding the applied bioassays are shown.

Supplementary Figure S7: Scans of cress seedlings treated with ecotoxic substances

This figure shows example scans of cress treated with the different ecotoxic substances cadmium nitrate, copper sulphate, iron sulphate, lead nitrate, manganese chloride, sodium chloride, zinc chloride, and with purified water as control. Only scans of seedlings treated with the five highest concentrations of the ecotoxic substances from 0.01 mM to 100 mM are shown in this figure because there were no changes in seedling growth in solutions with lower concentrations. The scale bar represents 1 cm.
