# Supplementary material for: A versatile Lepidium sativum bioassay for use in ecotoxicological studies
Source: Sci Rep. 2025 Sep 23;15:32653. doi: 10.1038/s41598-025-17215-7 (PMC12457589; doi:10.1038/s41598-025-17215-7)
Supplement: Supplementary file 3 — Legend Supplementary Video 1 [file 41598_2025_17215_MOESM3_ESM.pdf]

Legend Supplementary Video S1

Journal "Scientific Reports"

A versatile *Lepidium sativum* bioassay for use in ecotoxicological studies

Viola Maria Schulz, Claudia Scherr, Stephan Baumgartner and Alexander Tournier

Address correspondence to: Viola Schulz, MSc, Institute of Integrative Medicine, University of Witten/Herdecke, Gerhard-Kienle-Weg 4, 58313 Witten, Germany.

E-mail: Viola.Schulz@uni-wh.de

Supplementary Video S1: Video of aligning the cress seeds

This video shows how to easily arrange the cress seeds in a row after they have formed a mucus layer and before the growth period.
