# Supplementary material for: A versatile Lepidium sativum bioassay for use in ecotoxicological studies
Source: Sci Rep. 2025 Sep 23;15:32653. doi: 10.1038/s41598-025-17215-7 (PMC12457589; doi:10.1038/s41598-025-17215-7)
Supplement: Supplementary file 4 — Supplementary material 2 [file 41598_2025_17215_MOESM4_ESM.pdf]

Supplementary Method S2:

Manual for measurement procedure of seedling length with tablet and ImageJ

Journal "Scientific Reports"

# A versatile *Lepidium sativum* bioassay for use in ecotoxicological studies

Viola Maria Schulz, Claudia Scherr, Stephan Baumgartner and Alexander Tournier

Address correspondence to: Viola Schulz, MSc, Institute of Integrative Medicine, University of Witten/Herdecke, Gerhard-Kienle-Weg 4, 58313 Witten, Germany.

E-mail: Viola.Schulz@uni-wh.de

## Manual to measure seedling length
## with Windows Surface Tablet and ImageJ with plugin "Cress Measure Tool"

### Scanning of the seedlings

The bags containing the seedlings are scanned with the scanner EPSON Perfection V600 with the software 'Epson Scan' (Seiko Epson Corporation, Suwa, Nagano, Japan). To get a useful image quality, the following settings in the scanning software 'Epson Scan' are suitable: Full colour, photo, 600x600dpi, auto format B5, jpg, density medium without automatic, options: Sharpness as high as possible, A4 paper, copy factor 180%, density standard (no tick on automatic), not double-sided. The bags were gently and evenly pressed to the scanner glass with a plate from above to prevent the seedlings from bulging. Millimetre paper was scanned alongside each bag to allow calibration in the subsequent analysis.

Measuring seedling length

Seedlings on scans can be tracked with the help of the digital tablet Windows Surface Pro 2017 (Microsoft, Redmont, Washington, United States of America) and the software ImageJ (version 1.52n with Java 1.8.0_112, 64-bit) (Collins, 2007) with the plugin “Cress Measure Tool”. A tablet pen is needed to track the seedlings, for example Wacom Bamboo Ink CS-321. The software and the associated plugin allow a true curve length measurement of the seedlings. The seedling length is measured manually by drawing a line in accordance with the shape of the seedling. To start detecting of the seedling length, before or after opening the scans of the seedlings, the plugin ‘Cress Measure Tool’ (provided in Online Resource 4) is installed. This plugin was created based on the ImageJ plugin "Segmented Freehand Line Tool" by Jan Eglinger, which was then modified in a way that makes it possible to add a line to the ROI manager with a chosen key on the keyboard without lifting the pen (in this study, ‘y’ was used). A detailed description of the plugin’s functions can be found at the end of this text. Under ‘File’ and ‘Open’, a scan can be opened. To open a series of scans, choose ‘File’, ‘Import’ and ‘Image Sequence’. Therefore, all scans that should be opened need to be collected in the same folder. To open the ROI manager, it can be found in menu ‘Analyze’, ‘Tools’, ‘ROI Manager’. (It also opens automatically while measuring by pressing the chosen key.) It is helpful to make a tick in the ROI Manager at ‘Show All’ and ‘Labels’ to make the drawn lines and their labels visible.

Calibration is done at the start of each measurement valid for all images that have been opened as a series. A calibration line with a chosen length can be set for each experiment by the help of the millimetre paper on the scans. Calibration with this line can be done with ImageJ function ‘Set Scale’ (menu ‘Analyze’). The ROIs of the calibration line can be saved with the ROI manager to be able to continue the

measurement of the seedlings of the same experiment with the same calibration later on. Before measuring, the images should be opened as large as possible.

It is helpful to zoom in to 50% (menu ‘Image’, ‘Zoom’, ‘Set…’, ‘Zoom: 50%’) which allows a better visual identification of the seedlings. The scrolling tool can be used to correct the position of the scans in the opened image so that all seedlings are visible.

By using the tablet pen, a line can then be drawn over the seedlings on the scans to measure their length. At the beginning, a short single line is drawn somewhere on the image to assure to be in the correct window, otherwise an error message could occur later in the drawing process. It is necessary to double type with the pen at this starting point otherwise the measurement will start with a little delay and the line will not be exact. The value of the line drawn before double typing with the pen will be duplicated in the results list. This redundant value can be easily removed using the Excel template, into which the list of measurement values can be inserted. After double typing, the seedling can be tracked with drawing a line along its shape. At the transition of shoot and root, the chosen key (in this study, ‘y’ was used) can be pressed to add this line to the ROI manager without lifting the pen and to immediately start drawing. This leads to the detection of the shoot length with the first part of the line. The root can then be tracked by drawing the newly started line according to its shape until its end. The measurement ends by lifting the pen at the end of the root and subsequently, the total length of the line/seedling is measured.

As a next step, the lines can be checked for their completeness and accuracy with the help of the ROI manager by clicking on the values. The chosen line is shown in a different colour than other lines which allows to check the accuracy of the line at the transition of shoot and root. Values obtained can be seen under ‘Measure’. Afterwards, all of the values can be selected, copied and pasted in a prepared Excel template that removes unnecessary values like the first line and calculates root length and root to

shoot ratio with the measured values for shoot length and total length. In this prepared excel file, all values are collected automatically in an Excel workbook. Values for date and number of seedlings and other information can easily be added in this template and the list can quickly be prepared for statistical analysis. In addition, the text values of the measured lines and the ROIs can be saved in ImageJ.

| Detailed description of functions of the ImageJ plugin “Cress Measure Tool” | | |
|---|---|---|
| **Part 1** | **Recording the initial position** | |
| | var leftButton = 16;<br>var shift = 1;<br>var shiftChanged = 0;<br>getCursorLoc(x, y, z, flags);<br>xArr = newArray(1); xArr[0] = x;<br>yArr = newArray(1); yArr[0] = y; | Captures the current mouse or pen position and stores it in arrays (ordered collections of values stored under a common name) to subsequently draw a freehand line based on these coordinates. |
| **Part 2** | **Initiation of the infinite loop (drag mode)** | |
| | while (true) {<br>    getCursorLoc(x, y, z, flags); | Initiates an infinite loop to track the movements of the mouse or tablet pen while the button is held down or the pen remains in contact. |
| | **Case: Mouse button released / tablet pen lifted** | |
| | if (flags & leftButton == 0) {<br>    roiManager("Add");<br>    return;<br>} | When the tablet pen is lifted or the mouse button is released, the current line is finalised and added as Region of Interest (ROI) to the ROI Manager. |
| **Part 3** | **Continuing to draw** | |
| | xArr = Array.concat(xArr, x);<br>yArr = Array.concat(yArr, y);<br>makeSelection("freeline", xArr, yArr); | Draws the freehand line continuously by appending new mouse positions to the array (while the mouse button is held down or the tablet pen remains in contact and is moved). |
| | **Shift key is pressed or released** | |
| | if (flags & shift != shiftChanged)<br>{<br>    roiManager("Add");<br>    xArr = newArray(1); xArr[0] = x;<br>    yArr = newArray(1); yArr[0] = y;<br>    shiftChanged = flags & shift;<br>} | If the Shift key is pressed or released, the current line is finalised and appended to the ROI Manager. Concurrently, a new line is initiated automatically. (This functionality is not strictly essential when operating solely with a tablet pen, but it may represent a beneficial feature if required.) |

| | | |
|---|---|---|
| | **Brief pause** | |
| | wait(10); | Brief pause to reduce CPU load, which enables smoother tracking. |
| **Part 4** | **Manual addition of an ROI using the y key** | |
| | macro "Add [y]" {<br>roiManager ("add");<br>} | Defines a keyboard shortcut (y) to manually add ROIs. This allows a line to be completed and a new one started without lifting the tablet pen. |

.
