# Supplementary material for: A versatile Lepidium sativum bioassay for use in ecotoxicological studies
Source: Sci Rep. 2025 Sep 23;15:32653. doi: 10.1038/s41598-025-17215-7 (PMC12457589; doi:10.1038/s41598-025-17215-7)
Supplement: Supplementary file 6 — Legend Supplementary Video 3 [file 41598_2025_17215_MOESM6_ESM.pdf]

Legend Supplementary Video S3

Journal "Scientific Reports"

A versatile *Lepidium sativum* bioassay for use in ecotoxicological studies

Viola Maria Schulz, Claudia Scherr, Stephan Baumgartner and Alexander Tournier

Address correspondence to: Viola Schulz, MSc, Institute of Integrative Medicine, University of Witten/Herdecke, Gerhard-Kienle-Weg 4, 58313 Witten, Germany.

E-mail: Viola.Schulz@uni-wh.de

Supplementary Video S3: Video of the length measurement procedure with the digital tablet and ImageJ

This video demonstrates the measurement procedure for the scan of one bag containing cress seedlings, illustrating how seedling length is determined using the Windows surface tablet in combination with the software ImageJ and an associated plugin.
