# Supplementary material for: A versatile Lepidium sativum bioassay for use in ecotoxicological studies
Source: Sci Rep. 2025 Sep 23;15:32653. doi: 10.1038/s41598-025-17215-7 (PMC12457589; doi:10.1038/s41598-025-17215-7)
Supplement: Supplementary file 7 — Supplementary File 4 [file 41598_2025_17215_MOESM7_ESM.docx]

Supplementary File S4:
ImageJ Plugin “Cress Measure Tool”

Journal "Scientific Reports"
**A versatile *Lepidium sativum* bioassay for use in ecotoxicological studies**

Viola Maria Schulz, Claudia Scherr, Stephan Baumgartner and Alexander Tournier
Address correspondence to: Viola Schulz, MSc, Institute of Integrative Medicine, University of Witten/Herdecke, Gerhard-Kienle-Weg 4, 58313 Witten, Germany.
E-mail: [Viola.Schulz@uni-wh.de](mailto:Viola.Schulz@uni-wh.de)

The following text constitutes the ImageJ plugin that enables curve length measurements of seedlings. It also allows lines to be drawn separately for the shoot and root without lifting the pen, by pressing a defined key on the keyboard at the point where the shoot ends and the root begins. This plugin was developed based on the ImageJ plugin "Segmented Freehand Line Tool" by Jan Eglinger.

macro Cress Measure Tool -

{

var leftButton = 16;

var shift = 1;

var shiftChanged = 0;

getCursorLoc(x, y, z, flags);

xArr = newArray(1); xArr[0] = x;

yArr = newArray(1); yArr[0] = y;

// drag mode

while (true) {

getCursorLoc(x, y, z, flags);

if (flags&leftButton==0) {

// button released - add last selection

roiManager("Add");

return;

}

xArr = Array.concat(xArr,x);

yArr = Array.concat(yArr,y);

makeSelection("freeline", xArr, yArr);

if (flags&shift!=shiftChanged) {

// shift key changed - add selection

roiManager("Add");

xArr = newArray(1); xArr[0] = x;

yArr = newArray(1); yArr[0] = y;

shiftChanged = flags&shift;

}

wait(10);

}

}

macro "Add [y]" {

roiManager ("add") ;

}
