# Supplementary material for: A versatile Lepidium sativum bioassay for use in ecotoxicological studies
Source: Sci Rep. 2025 Sep 23;15:32653. doi: 10.1038/s41598-025-17215-7 (PMC12457589; doi:10.1038/s41598-025-17215-7)
Supplement: Supplementary file 9 — Legend for Supplementary Material 5 [file 41598_2025_17215_MOESM9_ESM.pdf]

Legend for Supplementary File S5:

Excel file for seedling length analyses

Journal ‘Scientific Reports’

A versatile *Lepidium sativum* bioassay for use in ecotoxicological studies

Viola Maria Schulz, Claudia Scherr, Stephan Baumgartner and Alexander Tournier

Address correspondence to: Viola Schulz, MSc, Institute of Integrative Medicine, University of Witten/Herdecke, Gerhard-Kienle-Weg 4, 58313 Witten, Germany.

E-mail: Viola.Schulz@uni-wh.de

This Excel file provides a template for entering the data obtained from ImageJ measurements of cress seedlings, enabling straightforward calculation of the outcome parameters: shoot length, root length, total length, and root/shoot ratio.

Sheet 1 (‘Overview’) in the Excel file serves as overview table of the results. Information such as ‘Experiment number’, ‘Date’, ‘Substance’, ‘Concentration’, and ‘Amounts of seedlings’ can be entered manually in the first row and will then be applied to the entire table. The columns ‘Bag number’ and ‘Seedling number’ are pre-filled. A list with data of the evaluation parameters is automatically retrieved from the further Excel sheets corresponding to the individual bags. In these sheets, measurement data from seedling length measurements conducted using tablet and ImageJ can be pasted into cell B2. Any redundant values are excluded from further analysis. The measurement values for shoot length, total length, root length, and root/shoot ratio are calculated and listed. The list of these values is then automatically transferred to the summary table in sheet 1.
