# Supplementary material for: A versatile Lepidium sativum bioassay for use in ecotoxicological studies
Source: Sci Rep. 2025 Sep 23;15:32653. doi: 10.1038/s41598-025-17215-7 (PMC12457589; doi:10.1038/s41598-025-17215-7)
Supplement: Supplementary file 10 — Supplementary Material 6 [file 41598_2025_17215_MOESM10_ESM.pdf]

Supplementary List S6:

List of publications describing bioassays with garden cress found in PubMed and Web of Science (years 2010 - 2024)

Journal "Scientific Reports"

A versatile *Lepidium sativum* bioassay for use in ecotoxicological studies

Viola Maria Schulz, Claudia Scherr, Stephan Baumgartner, and Alexander Tournier

Address correspondence to: Viola Schulz, MSc, Institute of Integrative Medicine, University of Witten/Herdecke,

Gerhard-Kienle-Weg 4, 58313 Witten, Germany. E-mail: Viola.Schulz@uni-wh.de

List of publications describing bioassays with garden cress as test organism
– extracted from PubMed and Web of Science (years 2010-8/2020 and update search 9/2020-9/2024)

No.	Year	First author	Publication title	Growth container	Measurement method (- = not applicable; N/A = not stated)	References for bioassay
1	2010	De Almeida	Phytotoxic activities of mediterranean essential oils	Petri dish	N/A	
2	2010	De Martino	Chemical composition and antigerminative activity of the essential oils from five *Salvia*	Petri dish	N/A	

3	2010	Ellouze	Study on the influence of high salts content on fungal treatment of saline wastewaters	N/A	-	Zucconi *et al.,* 1981
4	2010	Gouider	Bioassay and use in irrigation of untreated and treated wastewaters from phosphate fertilizer industry	Petri dish	N/A	Zucconi *et al.,* 1981
5	2010	Kato-Noguchi	Allelopathy and allelopathic substance in the moss *Rhynchostegium pallidifolium*	Petri dish	N/A	
6	2010	Kato-Noguchi	Isolation and identification of potent allelopathic substances in rattail fescue	Petri dish	N/A	
7	2010	Krome	Soil bacteria and protozoa affect root branching via effects on the auxin and cytokinin balance in plants	Petri dish	N/A	
8	2010	Luan	Maturity evaluation of spent mushroom compost	Petri dish	N/A	
9	2010	Luan	Composts from green waste as peat substitutes for cutting propagation of *Impatiens hawkeri*	Petri dish	N/A	
10	2010	Muhammad	Vegetative growth performance of five medicinal plants under NaCl salt stress	pot	-	
11	2010	Muhammad	Effect of NaCl salinity on the germination and seedling growth of some medicinal plants	Petri dish	N/A	
12	2010	Nalcaci	Metal contamination characteristics of *Lepidium sativum* in a phosphate-, saline-, and nitrate-contaminated media	beaker	N/A	
13	2010	Oleszczuk	Toxicity of light soil fertilized by sewage sludge or compost in relation to PAHs content	test plate	software "Image Tool 3.0"	Phytotoxkit

14	2010	Paradelo	Evaluation of the toxicity of heavy metals and organic compounds in compost by means of two germination-elongation tests	Petri dish	N/A	Zucconi *et al.,* 1981
15	2010	Piotrowicz-Cieslak	Different glyphosate phytotoxicity of seeds and seedlings of selected plant species	test plate	software "Image Tool"	Phytotoxkit™ (MicroBio Test Inc., Belgium)
16	2010	De Martino	Chemical composition and phytotoxic effects of essential oils from four *Teucrium* species	Petri dish	N/A	
17	2010	De Martino	The antigerminative activity of twenty-seven monoterpenes	Petri dish	N/A	
18	2010	Jones	Effect of amendment of bauxite processing sand with organic materials on its chemical, physical and microbial properties	Petri dish	-	
19	2010	Saddoud	A comparative study of the industrial discharges effect on the anaerobic treatment of domestic wastewater in both experimental and pilot-plant scales	N/A	-	Zucconi *et al.*, 1981
20	2011	Banaszkiewicz	Biological evaluation of soil contamination around a non-operating pesticide tomb	special transparent vessels	N/A	Phytotoxkit; Oleszczuk 2008
21	2011	Del Buono	Utilization of olive husks as plant growing substrates: phytotoxicity and plant biochemical responses	N/A	N/A	Zucconi *et al.,* 1985
22	2011	Kato-Noguchi	Isolation and identification of potent allelopathic substances in a traditional Bangladeshi rice cultivar Kartikshail	Petri dish	N/A	

23	2011	Kato-Noguchi	Two potent allelopathic substances in cucumber plants	Petri dish	N/A	
24	2011	Morris	Regulation of seed germination in the close Arabidopsis relative *Lepidium sativum*: a global tissue-specific transcript analysis	Petri dish	binocular microscope	
25	2011	Rakleviciene	Clinorotation effect on response of cress leaves to red and far-red light	centrifuge-clinostat/metallic containers for plant cultivation		
26	2011	Roca-Pérez	Assessing compost phytotoxicity using compost eluates and a compost plate bioassay	Petri dish	N/A	Zucconi *et al.,* 1985; Boluda *et al.*, 2011
27	2011	Shehzad	Effect of weed-crop competition on growth and yield of garden cress (*Lepidium sativum* L.)	field	-	
28	2011	Svegzdiene	Comparison study of gravity-dependent displacement of amyloplasts in statocytes of cress roots and hypocotyls	centrifuge-clinostat/metallic containers for plant cultivation		
29	2011	Szakiel	Isolation and biological activities of lyoniside from rhizomes and stems of *Vaccinium myrtillus*	Petri dish	N/A	
30	2011	Bakopoulou	Assessment of wastewater effluent quality in thessaly region, greece, for determining its irrigation reuse potential	test plates	software "Image Tool 3.0"	Phytotoxkit protocol, Microbiotests Inc., Biohidrica Ltd: Phytotoxkit Bench Protocol

31	2011	Ceccanti	Phytotreatment of sludges (*Phragmites Australis*) for their reuse in the environment	plastic pots	-	
32	2011	Czerniawska-Kusza	The potential of the Phytotoxkit microbiotest for hazard evaluation of sediments in eutrophic freshwater ecosystems	N/A	N/A	Phytotoxkit, 2004
33	2011	Gargouri	Application of a continuously stirred tank bioreactor (CSTR) for bioremediation of hydrocarbon-rich industrial wastewater effluents	N/A	-	Zucconi *et al.*, 1985
34	2011	Gómez-Muñoz	Carbon mineralization and distribution of nutrients within different particle-size fractions of commercially produced olive mill pomace	Petri dish	N/A	Zucconi *et al.*, 1981
35	2011	Graeber	A guideline to family-wide comparative state-of-the-art quantitative RT-PCR analysis exemplified with a Brassicaceae cross-species seed germination case study	Petri dish	-	Linkies *et al.*, 2009
36	2011	Himanen	Composting of bio-waste, aerobic and anaerobic sludges – effect of feedstock on the process and quality of compost	Petri dish, plates	N/A	
37	2011	Jones	Influence of organic waste and residue mud additions on chemical, physical and microbial properties of bauxite residue sand	Petri dish	N/A	
38	2011	Mancini	Chemical composition and possible *in vitro* phytotoxic activity of *Helichrysum italicum* (Roth) Don *ssp. italicum*	Petri dish	N/A	
39	2011	Manzo	Investigation of ZnO nanoparticles' ecotoxicological effects towards different soil organisms	N/A	N/A	EPA, 1996; OECD, 2003

40	2011	Marandino	Chemical composition and possible *in vitro* antigermination activity of three hypericum essential oils	Petri dish	N/A	
41	2011	Michalczyk	Mycetozoal bloom in a hydroponic culture of garden cress (*Lepidium sativum* L.)	plastic plates	-	
42	2011	Morris	Regulation of seed germination in the close arabidopsis relative *Lepidium sativum*: A global tissue-specific transcript analysis	Petri dish	-	
43	2011	Negre	Changes in chemical and biological parameters during co-composting of anaerobically digested sewage sludges with lignocellulosic material	N/A	-	
44	2011	Oleszczuk	Comparison of sewage sludge toxicity to plants and invertebrates in three different soils	test plates	software "Image Tool 3.0"	Phytotoxkit (MicroBioTests, Nazareth, Belgium)
45	2011	Oleszczuk	The toxicity to plants of the sewage sludges containing multiwalled carbon nanotubes	test plates	software "Image Tool 3.0"	Phytotoxkit (MicroBioTests, Nazareth, Belgium)
46	2011	Rahmann	Aquatic arsenic: Phytoremediation using floating macrophytes	N/A	-	
47	2011	Ruggiero	Hg bioavailability and impact on bacterial communities in a long-term polluted soil	Petri dish	N/A	
48	2011	Voegele	Members of the gibberellin receptor gene family GID1 (Gibberellin Insensitive DWARF1) play distinct roles during *Lepidium sativum* and *Arabidopsis thaliana* seed germination	Petri dish	-	

49	2012	Amri	Chemical composition and biological activities of the essential oils from three *Melaleuca species* grown in Tunisia	Petri dish	N/A	
50	2012	Barrientos	Impact of cadmium and selenium exposure on trace elements, fatty acids and oxidative stress in *Lepidium sativum*	in medium	-	
51	2012	Belloncle	Study of the degradation of poly(ethyl glyoxylate): biodegradation, toxicity and ecotoxicity assays	flask	-	
52	2012	Bystrzejewska-Piotrowska	Application of neutron activation for investigation of Fe_3O_4 nanoparticles accumulation by plants	containers	-	
53	2012	De Martino	Vitro phytotoxicity and antioxidant activity of selected flavonoids	Petri dish	N/A	
54	2012	Fornalski	Search of radiation hormesis in plants: irradiation of the cress (*Lepidium sativum* L.)	scale pan	N/A	
55	2012	Jankowiak	Root-colonizing ophiostomatoid fungi associated with dying and dead young Scots pine in Poland	flask	N/A	
56	2012	Kato-Noguchi	Organ-specific-active allelopathic substance in red pine needles	Petri dish	N/A	
57	2012	Kato-Noguchi	A potent allelopathic substance in cucumber plants and allelopathy of cucumber	Petri dish	N/A	
58	2012	Negre	Horticultural and floricultural applications of urban wastes originated fertilizers	on filter paper	N/A	
59	2012	Nicola	Nitrogen and aeration levels of the nutrient solution in soilless cultivation systems as important growing	N/A	-	Fontana and Nicola, 2008

			conditions affecting inherent quality of baby leaf vegetables: a review			
60	2012	Tran	Allelopathic potential of two aquatic plants, duckweed (*Lemna minor* L.) and water lettuce (*Pistia stratiotes* L.), on terrestrial plant species	Petri dish	N/A	
61	2012	Amri	Chemical composition and biological activities of the essential oils from three *Melaleuca* species grown in Tunisia	Petri dish	N/A	
62	2012	Baumgartner	Development of a biocrystallisation assay for examining effects of homeopathic preparations using cress seedlings	plastic bags	-	Baumgartner und Flückiger, 2001
63	2012	Busch	Simple biotoxicity tests for evaluation of carbonaceous soil additives: establishment and reproducibility of four test procedures	glass jars	-	
64	2012	De Martino	*In vitro* phytotoxicity and antioxidant activity of selected flavonoids	Petri glasses	N/A	
65	2012	Gill	Cadmium at high dose perturbs growth, photosynthesis and nitrogen metabolism while at low dose it up regulates sulfur assimilation and antioxidant machinery in garden cress (*Lepidium sativum* L.)	earthen pots	-	
66	2012	Himanen	Phytotoxicity of low-weight carboxylic acids	Petri dish	N/A	
67	2012	Kutschera	Root phototropism: from dogma to the mechanism of blue light perception	N/A	N/A	

68	2012	Michael	Solar photo-fenton process on the abatement of antibiotics at a pilot scale: degradation kinetics, ecotoxicity and phytotoxicity assessment and removal of antibiotic resistant enterococci	N/A	N/A	Phytotestkit microbiotest (Micro-BioTests Inc.)
69	2012	Müller	Role of a respiratory burst oxidase of *Lepidium sativum* (cress) seedlings in root development and auxin signalling	Petri dish	-	
70	2012	Oleszczuk	Influence of activated carbon and biocharon phytotoxicity of air-dried sewage sludges to *Lepidium sativum*	test plates	software "Image Tool 3.0"	Phytotoxkit™ Test (Microbiotests,Nazareth,Belgium)(Phytotoxkit, 2004).
71	2012	Oleszczuk	The phytotoxicity changes of sewage sludge-amended soils	test plates	software "Image Tool 3.0"	Phytotoxkit, 2004
72	2012	Oracz	Myrigalone A inhibits *Lepidium sativum* seed germination by interference with gibberellin metabolism and apoplastic superoxide production required for embryo extension growth and endosperm rupture	Petri dish	-	
73	2012	Russo	Ozone oxidation and aerobic biodegradation with spent mushroom compost for detoxification and benzo(a)pyrene removal from contaminated soil	Petri dish	N/A	OECD, 2006; USEPA, 1991
74	2012	Voegele	Embryo growth, testa permeability, and endosperm weakening are major targets for the environmentally regulated inhibition of *Lepidium sativum* seed ermination by myrigalone A	Petri dish	software "ImageJ"	

75	2013	Amri	Chemical composition and biological activities of the essential oil from *Artemisia herba-alba* growing wild in Tunisia	Petri dish	N/A
76	2013	Andrianjafinandrasana	Allelopathic effects of volatile compounds of essential oil from *Ravensara aromatica* Sonnerat chemotypes	glass jar	-
77	2013	Bargmann	Hydrochar and biochar effects on germination of spring barley	Petri dish	N/A
78	2013	Barrientos	Application of reversed-phase high-performance liquid chromatography with fluorimetric detection for simultaneous assessment of global DNA and total RNA methylation in *Lepidium sativum*: effect of plant exposure to Cd(II) and Se(IV)	in medium	-
79	2013	El-Darier	Differential allelopathic effect of nine *Haplophyllum tuberculatum* growth forms through germination bioassay	Petri dish	N/A
80	2013	Luo	Al aromatic moieties from matured chicken manure and agriculture residues compost suppress growth of *Lepidium sativum* L. and *Trichoderma harzianum*	Petri dish	N/A
81	2013	Pave	Effects of heavy metals on *Lepidium sativum* germination and growth	Petri dish	N/A
82	2013	Saadi	Fate of soil-applied olive mill wastewater and potential phytotoxicity assessed by two bioassay methods	square Petri dish	N/A
83	2013	Suherman	Partitioning of copper and lead between solid and dissolved organic matter in a humus-rich soil of the	Petri dish	ruler

			Harz Mountains (Germany) and ecotoxicity test with *Lepidium sativum*			
84	2013	Tanabe	Palmaerins A-D, new chlorinated and brominated dihydroisocoumarins with antimicrobial and plant growth regulating activites from discomycete *Lachnum Palmae*	Petri dish	N/A	
85	2013	Baran	Phytotoxkit/Phytotest kit and Microtox as tools for toxicity assessment of sediments	test plates	photos taken with digital camera and analysed with software "Image Tools"	Phytotoxkit, 2004
86	2013	Bedell	Assessment of the phytotoxicity of seaport sediments in the framework of a quarry-deposit scenario: Germination tests of sediments aged artificially by column leaching	micro-plates	-	Phytotoxkit F™; Phytotoxkit, 2004; R-Biopharm, France [2004], OECD 2006
87	2013	Buerge	The chiral herbicide beflubutamid (I): Isolation of pure enantiomers by HPLC, herbicidal activity of enantiomers, and analysis by enantioselective GC-MS	pipet tips with agar	N/A	
88	2013	Choi	First report of powdery mildew caused by *Erysiphe cruciferarum* on garden cress in Korea	pots	-	
89	2013	Frascari	Aerobic/anaerobic/aerobic sequenced biodegradation of a mixture of chlorinated ethenes, ethanes and methanes in batch bioreactors	N/A	N/A	Bertin *et al.,* 2007; Kreysa and Wiesner, 1995
90	2013	Gómez Ojeda	Effect of Cd(II) and Se(IV) exposure on cellular distribution of both elements and concentration levels of glyoxal and methylglyoxal in *Lepidium sativum*	N/A	-	

91	2013	Jósko	Effect of biochars, activated carbon and multiwalled carbon nanotubes on phytotoxicity of sediment contaminated by inorganic and organic pollutants	test plate	software "Image Tool 3.0"	Phytotoxkit F™; Phytotoxkit, 2004
92	2013	Jósko	Influence of soil type and environmental conditions on ZnO, TiO_2 and Ni nanoparticles phytotoxicity	test plates	N/A	Phytotoxkit™ Test; Phytotoxkit, 2004
93	2013	Jósko	The influence of ZnO and TiO_2 nanoparticles on the toxicity of sewage sludges	test plates	software "Image Tool 3.0"	Phytotoxkit™ Test; Phytotoxkit, 2004; OECD, 2003
94	2013	Kato-Noguchi	A novel allelopathic substance, 13-epi-orthosiphol N, in *Orthosiphon stamineus*	Petri dish	N/A	2013
95	2013	Kato-Noguchi	Allelopathy is involved in the formation of pure colonies of the fern *Gleichenia japonica*	Petri dish	N/A	
96	2013	Kato-Noguchi	A novel substance with allelopathic activity in *Ginkgo biloba*	Petri dish	N/A	
97	2013	Keser	Effects of irrigation with wastewater on the physiological properties and heavy metal content in *Lepidium sativum* L. and *Eruca sativa* (Mill.)	plastic pots	N/A	
98	2013	Mancini	Chemical composition and biological activities of the essential oil from *Calamintha nepeta* plants from the wild in southern Italy	Petri dish	N/A	
99	2013	Oleszczuk	The toxicity to plants of the sewage sludges containing multiwalled carbon nanotubes	test plates	software "Image Tool 3.0"	Phytotoxkit, 2004
100	2013	Umar	Arsenic toxicity in garden cress (*Lepidium sativum* Linn.): significance of potassium nutrition	Petri dish	meter scale	

101	2013	Visioli	Metal toxicity and biodiversity in serpentine soils: Application of bioassay tests and microarthropod index	Petri dish	N/A	OECD, 1984
102	2013	Yanez	Application of reversed-phase high-performance liquid chromatography with fluorimetric detection for simultaneous assessment of global DNA and total RNA ethylation in *Lepidium sativum*: effect of plant exposure to Cd(II) and Se(IV)	N/A	-	
103	2014	Islam	Phytotoxic activity of *Ocimum tenuiflorum* extracts on germination and seedling growth of different plant species	Petri dish	N/A	
104	2014	Kato-Noguchi	Growth limiting effects on various terrestrial plant species by an allelopathic substance, loliolide, from water hyacinth	Petri dish	N/A	
105	2014	Kashkooli	Essential oil compositions and natural herbicide activity of four denaei thyme (*Thymus daenensis* Celak.) ecotypes	Petri dish	N/A	
106	2014	Manaa	Superoxide dismutase isozyme activity and antioxidant responses of hydroponically cultured *Lepidium sativum* L. to NaCl stress	peat	N/A	
107	2014	Montvydiene	Impact of closed Kairiai landfill on the Ginkunai Pond	Petri dish	N/A	
108	2014	Nebo	Phytotoxicity of alkaloids, coumarins and flavonoids isolated from 11 species belonging to the Rutaceae and Meliaceae families	Petri dish	N/A	Macias *et al.,* 2000

109	2014	Onaran	Antifungal and bioherbicidal properties of essential oils of *Thymus fallax* Fish & Mey, *Origanum vulgare* L. and *Mentha dumetorum* Schult	Petri dish	N/A	
110	2014	Perveen	Extraction, isolation, and identification of various environment friendly components from cock's comb (*Celosia argentea*) leaves for allelopathic potential	Petri plate	N/A	
111	2014	Agnieszka	Chemical properties and toxicity of soils contaminated by mining activity	N/A	N/A	Phytotoxkit, 2004
112	2014	Baderna	Soil quality in the Lomellina area using *in vitro* models and ecotoxicological assays	Petri dish	N/A	Martignon, 2009
113	2014	Baumgartner	Evaluation of preclinical assays to investigate an anthroposophic pharmaceutical process applied to mistletoe (*Viscum album* L.) extracts	plastic bags	graphics tablet connected to an Apple Macintosh computer using special software	
114	2014	Bich	Isolation and identification of a phytotoxic substance from the emergent macrophyte *Centrostachys aquatica*	Petri dish	N/A	
115	2014	Buss	Mobile organic compounds in biochar - a potential source of contamination - phytotoxic effects on cress seed (*Lepidium sativum*) germination	plastic jar	N/A	
116	2014	Chevillard	Biodegradable herbicide delivery systems with slow diffusion in soil and UV protection properties	polystyrene square containers	-	
117	2014	Del Moro	Landfill wall revegetation combined with leachate recirculation: a convenient procedure for management of closed landfills	Petri dish	measuring tape	EPA Guidelines

118	2014	Deravel	Mycosubtilin and surfactin are efficient, low ecotoxicity molecules for the biocontrol of lettuce downy mildew	Petri dish	N/A	
119	2014	Di Maria	Co-treatment of fruit and vegetable waste in sludge digesters. An analysis of the relationship among bio-methane generation, process stability and digestate phytotoxicity	Petri dish	N/A	Pullicino *et al*., 2007; Gigliotti *et al*., 2012
120	2014	Fierro	Valorisation of used cooking oil sludge by codigestion with swine manure	Petri dish	N/A	Zucconi *et al*., 1981
121	2014	Gondek	The effect of low-temperature transformation of mixtures of sewage sludge and plant materials on content, leachability and toxicity of heavy metals	test plates	image was registered with a digital camera and software "Image Tools"	Phytotoxkit, 2004
122	2014	Graeber	DELAY OF GERMINATION 1 mediates a conserved coatdormancy mechanism for the temperature- and gibberellin-dependent control of seed germination	Petri dish	-	
123	2014	Islam	Phytotoxic activity of *Ocimum tenuiflorum* extracts on germination and seedling growth of different plant species	Petri dish	N/A	
124	2014	Islam	Two novel phytotoxic substances from *Leucas aspera*	Petri dish	N/A	Islam and Kato-Noguchi, 2013
125	2014	Islam	Suaveolic acid: A potent phytotoxic substance of *Hyptis suaveolens*	Petri dish	N/A	
126	2014	Jósko	Phytotoxicity of nanoparticles - problems with bioassay choosing and sample preparation	Petri dish, plates	software "Image Tool 3.0"	OECD, 1984; Phytotestkit F™ (MicroBioTests Inc., Belgium),

						Phytotoxkit F™ (MicroBioTests Inc., Belgium)
127	2014	Kato-Noguchi	Phytotoxic substances with allelopathic activity may be central to the strong invasive potential of *Brachiaria brizantha*	Petri dish	N/A	
128	2014	Kudlak	Ranking of ecotoxicity tests for underground water assessment using the Hasse diagram technique	N/A	N/A	Phytotoxkit F™
129	2014	Le Thi	Isolation and identification of an allelopathic phenylethylamine in rice	Petri dish	N/A	
130	2014	Li	Phytotoxic cis-clerodane diterpenoids from the Chinese liverwort *Scapania stephanii*	Petri dish	N/A	
131	2014	Mancini	Chemical composition and biological activity of the essential oil l of *Origanum vulgare ssp. hirtum* from different areas in the Southern Apennines (Italy)	Petri dish	N/A	
132	2014	Michael	Utilizing solar energy for the purification of olive mill wastewater using a pilot-scale photocatalytic reactor after coagulation-flocculation	N/A	N/A	Phytotestkit microbiotest (MicroBioTests Inc.)
133	2014	Tuhy	The application of biosorption for production of micronutrient fertilizers based on waste biomass	Petri dish	-	
134	2014	Ulukanli	Chemical composition, antimicrobial, insecticidal, phytotoxic and antioxidant activities of mediterranean *Pinus brutia* and *Pinus pinea* resin essential oils	Petri dish	N/A	
135	2014	Visioli	Germination and root elongation bioassays in six different plant species for testing Ni contamination in soil	Petri dish	N/A	

136	2015	Kato-Noguchi	Phytotoxicities of the invasive species *Plantago major* and non-invasive species *Plantago asiatica*	Petri dish	N/A	
137	2015	Kimura	Allelopathy of pine litter: Delivery of allelopathic substances into forest floor	Petri dish	N/A	
138	2015	Adamcová	Ecotoxicity of composts containing aliphatic-aromatic copolyesters	pot	-	ČSN EN13432
139	2015	Blicharska	High-resolution continuum source atomic absorption spectrometry with microwave-assisted extraction for the determination of metals in vegetable sprouts	beaker	-	
140	2015	Buchmann	Development of phytotoxicity and composition of a soil treated with olive mill wastewater (OMW): an incubation study	Petri dish	N/A	
141	2015	Fahad	Weed growth and crop yield loss in wheat as influenced by row spacing and weed emergence times	field / seed bed	N/A	
142	2015	Kocaçaliskan	Effects of walnut leaf extracts prepared in different solvents on seed germination and seedling growth of cress (*Lepidium sativum cv. Zeybek*)	Petri dish	N/A	
143	2015	Koltowski	Toxicity of biochars after polycyclic aromatic hydrocarbons removal by thermal treatment	N/A	N/A	Phytotoxkit F™; Phytotoxkit, 2004
144	2015	Meena	Effect of sowing durations and planting geometries on growth, productivity and quality of garden cress (*Lepidium sativum*)	field	-	
145	2015	Michalak	Seaweed extract by microwave assisted extraction as plant growth biostimulant	Petri dish	N/A	

146	2015	Nebo	Phytotoxicity of triterpenes and limonoids from the Rutaceae and Meliaceae	N/A	N/A	Nebo *et al.,* 2014
147	2015	Perveen	Extraction, isolation and structure confirmation of cock comb (*Celosia argentea)* leaves components for phytotoxic evaluation against *Lepidium sativum*	Petri dish	N/A	
148	2015	Ugolini	Production of an enzymatic protein hydrolyzate from defatted sunflower seed meal for potential application as a plant biostimulant	Petri dish	N/A	
149	2015	Asztemborska	Accumulation of platinum nanoparticles by sinapis alba and Lepidium sativum plants	containers	-	
150	2015	Aydin	Application of simple and low-Cost toxicity tests for ecotoxicological assessment of industrial wastewaters	Petri dish	N/A	Devare and Bahadir, 1994
151	2015	Baderna	Chemical characterization and ecotoxicity of three soil foaming agents used in mechanized tunneling	Petri dish	N/A	Martignon, 2009 with slight modifications as in Baderna *et al*., 2014
152	2015	Baderna	Acute phytotoxicity of seven metals alone and in mixture: Are Italian soil threshold concentrations suitable for plant protection?	Petri dish	N/A	UNICHIM 1651 guideline for phytotoxicity test (2003); Martignon, 2009 with slight modifications as in Baderna *et al*., 2014
153	2015	Buerge	Environmental behavior of the chiral herbicide haloxyfop. 2. unchanged enantiomer composition in blackgrass (*Alopecurus myosuroides*) and garden cress (*Lepidium sativum*)	glass vials	-	

154	2015	Choy	Co-composting of horticultural waste with fruit peels, food waste, and soybean residues	test plates	N/A	Phytotestkit (MicroBioTests Inc., Belgium)
155	2015	Demir	Degradation of a textile dye, Rhodamine 6G (Rh6G), by heterogeneous sonophoto Fenton process in the presence of Fe-containing TiO_2 catalysts	Petri dish	N/A	
156	2015	Fraternale	Essential oil composition and antigermination activity of *Artemisia dracunculus* (Tarragon)	Petri dish	N/A	
157	2015	Kato-Noguchi	A phytotoxic active substance in the decomposing litter of the fern *Gleichenia japonica*	Petri dish	N/A	
158	2015	Kobayashi	Phytotoxic substance with allelopathic activity in *Brachiaria decumbens*	Petri dish	N/A	
159	2015	Lipinska	Diversity of organotrophic bacteria, activity of dehydrogenases and urease as well as seed germination and root growth *Lepidium sativum*, *Sorghum saccharatum* and *Sinapis alba* under the influence of polycyclic aromatic hydrocarbons	plastic plates	N/A	Phytotoxkit
160	2015	Malakhova	The biotransformation of brewer’s spent grain into biogas by anaerobic microbial communities	pots	N/A	
161	2015	Michael-Kordatou	Erythromycin oxidation and ERY-resistant *Escherichia coli* inactivation in urban wastewater by sulfate radical-based oxidation process under UV-C irradiation	N/A	N/A	Phytotestkit (MicroBioTests Inc., Belgium); Michael *et al*., 2012
162	2015	Rombola	Relationships between chemical characteristics and phytotoxicity of biochar from poultry litter pyrolysis	Petri dish	-	

163	2015	Rózylo	An ecotoxicological evaluation of soil fertilized with biogas residues or mining waste	plastic pots	software "Image Tool 3.0"	Phytotoxkit F™; Phytotoxkit, 2004; OECD 1984
164	2015	Scheler	Promotion of testa rupture during garden cress germination involves seed compartment-specific expression and activity of pectin methyl esterases	Petri dish	-	
165	2015	Sciubba	Relationships between stability, maturity, water-extractable organic matter of municipal sewage sludge composts and soil functionality	Petri dish	-	
166	2015	Smolinska	Green waste compost as an amendment during induced phytoextraction of mercury-contaminated soil	plastic pots	N/A	Carrasco-Gil *et al.,* 2012
167	2015	Smolinska	Influence of combined use of iodide and compost on Hg accumulation by *Lepidium sativum* L.	plastic pots	-	
168	2015	Stefaniuk	Ecotoxicological assessment of residues from different biogas production plants used as fertilizer for soil	N/A	N/A	Phytotoxkit, 2004; Phytotestkit F
169	2015	Szklarek	The microbiotest battery as an important component in the assessment of snowmelt toxicity in urban watercourses - preliminary studies	N/A	-	Phytotoxkit™; Phytotoxkit, 2004
170	2016	Bozok	Volatiles from the aerial parts of east mediterranean clary sage: phytotoxic activity	Petri plate	N/A	
171	2016	Gheorghe	Phytotoxicity tests applied on sewage sludge resulted from urban wastewater treatment	on contaminat ed sludge	software "ImageTool"	Phytotoxkit microbiotest protocol which follows the ISO 11269-1:2012 methodology

172	2016	Grul'ová	Composition and bio activity of essential oils of *Solidago spp.*	Petri dish	N/A	
173	2016	Lencioni	Environmental application and phytotoxicity of anaerobic digestate from pig farming by *in vitro* and *in vivo* trials	Petri dish	N/A	Zucconi *et al*., 1985
174	2016	Masum	Allelopathic potential of indigenous Bangladeshi rice varieties	Petri dish	N/A	
175	2016	Michalak	Supercritical fluid extraction of algae enhances levels of biologically active compounds promoting plant growth	Petri dish	N/A	
176	2016	Michalak	Co-composting of algae and effect of the compost on germination and growth of *Lepidium sativum*	Petri dish	N/A	
177	2016	Nicola	Overview of a lab-scale pilot plant for studying baby leaf vegetables grown in soilless culture	styroform plate	N/A	
178	2016	Ogórek	Enzymatic activity of potential fungal plant pathogens and the effect of their culture filtrates on seed germination and seedling growth of garden cress (*Lepidium sativum* L.)	Petri dish	N/A	
179	2016	Palas	Heterogeneous photo Fenton-like oxidation of Procion Red MX-5B using walnut shell based green catalysts	Petri dish	N/A	
180	2016	Ríos	Screening brassicaceous plants as biofumigants for management of *Phytophthora cinnamomi* oak disease	Petri dish	-	

181	2016	Tintner	Germination and juvenile development of plants on municipal solid waste incineration (MSWI) slag	Petri dish	-	BMLFUW, 2001
182	2016	Wieczorek	The effect of tert-butylhydroquinone (TBHQ) on biodiesel bioremediation in soil samples inoculated with bacterial cells	plate	N/A	Phytotoxkit®
183	2016	Alcazar	Application of liquid chromatography/electrospray ionization ion trap tandem mass spectrometry for the evaluation of global nucleic acids: methylation in garden cress under exposure to CuO nanoparticles	N/A	-	
184	2016	Apostolico	Chemical composition, antibacterial and phytotoxic activities of *Peganum harmala* seed essential oils from five different localities in Northern Africa	Petri dish	N/A	
185	2016	Balaguer	Compostability assessment of nano-reinforced poly(lactic acid) films	flowerpots	-	
186	2016	Bettiol	Assessment of phenolic herbicide toxicity and mode of action by different assays	glass Petri dish	N/A	
187	2016	Buss	Risks and benefits of marginal biomass-derived biochars for plant growth	N/A	software "ImageJ"	Buss and Mašek (2014)
188	2016	Godlewska	Plant growth biostimulants based on different methods of seaweed extraction with water	Petri dish	N/A	
189	2016	Grul'ová	Phytotoxic and antibacterial activity of essential oil of new peppermint cultivar	Petri dish	N/A	
190	2016	Kato-Noguchi	A potent phytotoxic substance in *Aglaia odorata* LOUR	Petri dish	N/A	

191	2016	Kim	Enhancement of the initial growth rate of agricultural plants by using static magnetic fields	Petri dish	N/A	
192	2016	Koltowski	Effect of steamactivated biochar application to industrially contaminated soils on bioavailability of polycyclic aromatic hydrocarbons and ecotoxicity of soils	N/A	N/A	Phytotoxkit, 2004
193	2016	Libralato	Phytotoxicity of ionic, micro- and nano-sized iron in three plant species	Petri dish	N/A	Beltrami *etal*., 1999; OECD, 2006
194	2016	Lingott	Gadolinium-uptake by aquatic and terrestrial organisms-distribution determined by laser ablation inductively coupled plasma mass spectrometry	N/A	-	
195	2016	Lofrano	Photocatalytic degradation of the antibiotic chloramphenicol and effluent toxicity effects	N/A	N/A	OECD, 2006
196	2016	Lofrano	Emerging concern from short-term textile leaching: A preliminary ecotoxicological survey	N/A	N/A	OECD, 2006; Libralato *et al.,* 2016
197	2016	Marchand	Effect of *Medicago sativa* L. and compost on organic and inorganic pollutant removal from a mixed contaminated soil and risk assessment using ecotoxicological tests	plastic pots	-	ISO 11269-2 (ISO, 1995)
198	2016	Matei	Hygienization and control of *Diplodia seriata* fungus in vine pruning waste composting and its seasonal variability in open and closed systems	N/A	N/A	Zucconi *et al.*, 1981
199	2016	Pivato	Use of digestate from a decentralized on-farm biogas plant as fertilizer in soils	Petri dish	N/A	

200	2016	Stefaniuk	Addition of biochar to sewage sludge decreases freely dissolved PAHs content and toxicity of sewage sludge-amended soil	N/A	N/A	Phytotoxkit, 2004; Phytotestkit F
201	2016	Urbaniak	Potential for phytoremediation of PCDD/PCDF-contaminated sludge and sediments using Cucurbitaceae plants: A pilot study	-	N/A	Phytotoxkit™ test kit (Microbiotests Inc., Belgium)
202	2016	Visioli	Assessing biochar ecotoxicology for soil amendment by root phytotoxicity bioassays	Petri dish	digital gauge	OECD, 2003
203	2017	Boonmee	Allelopathic activity of *Acacia concinna* pod extracts	Petri dish	N/A	
204	2017	Islam	2-Methoxystypandrone, a potent phytotoxic substance in Rumex maritimus	Petri dish	N/A	
205	2017	Suwitchayanon	*N-Octanoyl* tyramine, a phytotoxic compound in the roots of *Cymbopogon nardus*	Petri dish	N/A	
206	2017	Kayanifard	Allelopathic analysis of four ecotypes of ajowan	Petri dish	N/A	
207	2017	Märkl	Effect of leached cement paste samples with different superplasticiser content on germination and initial root growth of white mustard (*Sinapis alba*) and cress (*Lepidium sativum*)	Petri dish	N/A	
208	2017	Michalak	Bioconversion of baltic seaweeds into organic compost	Petri dish	N/A	
209	2017	Pampuro	Phytotoxicity and chemical characterization of compost derived from pig slurry solid fraction for organic pellet production	Petri dish	N/A	
210	2017	Rombel-Bryzek	The impact of copper ions on oxidative stress in garden cress *Lepidium sativum*	Petri dish	-	

211	2017	Thi	Synthesis and plant growth inhibitory activity of N-*trans*-cinnamoyltyramine: its possible inhibition mechanisms and biosynthesis pathway	plate	N/A	Thi *et al.,* 2014b
212	2017	Godlewska	The influence of pH of extracting water on the composition of seaweed extracts and their beneficial properties on *Lepidium sativum*	Petri dish	N/A	
213	2017	Gomes	Photocatalytic ozonation using doped TiO_2 catalysts for the removal of parabens in water	Petri dish	N/A	
214	2017	Jósko	Toxicity of combined mixtures of nanoparticles to plants	Petri dish	software "Image Tool 3.0"	OECD, 1984
215	2017	Kato-Noguchi	Asparagus decline: autotoxicity and autotoxic compounds in asparagus rhizomes	Petri dish	N/A	
216	2017	Koltowski	Effect of biochar activation by different methods on toxicity of soil contaminated by industrial activity	N/A	N/A	Phytotoxkit F; Phytotoxkit, 2004
217	2017	Mekki	Study of heavy metal accumulation and residual toxicity in Soil Saturated with phosphate Processing Wastewater	N/A	N/A	Zucconi *et al.*, 1981
218	2017	Michalak	Characterisation of biological properties of co-composted Baltic seaweeds in germination tests	plates	-	
219	2017	Pantazopoulou	Stabilization of tannery sludge by co-treatment with aluminum anodizing sludge and phytotoxicity of end-products	test plates	software "Image Tool 3.0"	Phytotoxkit™ Test, 2004
220	2017	Pinho	Phytotoxicity assessment of olive mill solid wastes and the influence of phenolic compounds	Petri dish	N/A	

221	2017	Ricci	The essential oil of *Monarda didyma* L. (*Lamiaceae*) exerts phytotoxic activity *in vitro* against various weed seeds	Petri dish	N/A	De Martino, 2010
222	2017	Schulz	6-Hydroxy-5-nitrobenzo[d]oxazol-2(3H)-one - A degradable derivative of natural 6-Hydroxybenzoxazolin-2(3H)-one produced by *Pantoea ananatis*	N/A	N/A	Macias, 2005
223	2017	Smolinska	Antioxidative response of *Lepidium sativum* L. during assisted phytoremediation of Hg contaminated soil	N/A	-	
224	2017	Smolinska	Protein changes in *Lepidium sativum* L. exposed to Hg during soil phytoremediation	N/A	-	Smolinska and Rowe, 2015; Smolinska and Leszczynska, 2015
225	2017	Smolinska	Photosynthetic pigments and peroxidase activity of *Lepidium sativum* L. during assisted Hg phytoextraction	plastic pots	-	Smolinska and Leszczynska, 2015
226	2017	Sobariu	Rhizobacteria and plant symbiosis in heavy metal uptake and its implications for soil bioremediation	Petri dish	N/A	
227	2017	Sobik-Szoltysek	Effect of addition of sewage sludge and coal sludge on bioavailability of selected metals in the waste from the zinc and lead industry	Petri dish	N/A	
228	2017	Urbaniak	The effect of sewage sludge application on soil properties and willow (*Salix sp.*) cultivation	N/A	N/A	Phytotoxkit™ (Microbiotest Inc., Nazareth, Belgium); Phytotoxkit, 2004
229	2017	Urbaniak	The influence of the *Cucurbitaceae* on mitigating the phytotoxicity and PCDD/PCDF content of soil amended with sewage sludge	N/A	N/A	Phytotoxkit™ (Microbiotest Inc., Nazareth, Belgium); Phytotoxkit, 2004

230	2018	Zaman	Allelopathic property and an allelopathic substance in *Eleocharis atropurpurea* (Retz.)	Petri dish	N/A	
231	2018	Abyaneh	Low frequency electromagnetic field induced oxidative stress in *Lepidium sativum* L.	Petri dish	-	
232	2018	Ciesielczuk	Acute toxicity of experimental fertilizers made of spent coffee grounds	plate	software "ImageTool"	Phytotoxkit microbiotest
233	2018	Laskawiec	An evaluation of the phytotoxicity of filter backwash water coagulation products from a pool water system	Petri dish	N/A	Phytotoxkit®; Phytotoxkit, 2004
234	2018	Mañas	Phytotoxicity test applied to sewage sludge using *Lactuca sativa* L. seeds	Petri dish	N/A	
235	2018	Masum	Isolation and characterization of allelopathic compounds from the indigenous rice variety 'Boterswar' and their biological activity against *Echinochloa crus-galli* L	Petri dish	N/A	
236	2018	Noorhosseini	Improving seed germination and early growth of garden cress (*Lepidium sativum*) and basil (*Ocimum basilicum*) with hydro-priming	container	N/A	
237	2018	Pourbabaee	Degradation and detoxification of nicosulfuron by a *Pseudomonas* strain isolated from a contaminated cornfield soil	Petri dish	N/A	Tal *et al*., 2000
238	2018	Pribytkova	Sources of technogenic pollution influence on phytotoxicity of arctic soils	Petri dish	N/A	
239	2018	Roughani	Agro-morphological study on several accessions of garden cress in Iran	field	-	

240	2018	Schulz	*Pantoea ananatis* converts MBOA to 6-Methoxy-4-nitro-benzoxazolin-2(3H)-one (NMBOA) for cooperative degradation with its native root colonizing microbial consortium	Petri dish	-	
241	2018	Seggiani	Polycaprolactone-collagen hydrolysate thermoplastic blends: Processability and biodegradability/compostability	Petri dish	N/A	
242	2018	Smolinska	Activation of Non-Enzymatic Antioxidants by *Lepidium sativum* L. Exposed to Hg During Assisted Phytoextraction	pot	-	
243	2018	Souri	Effects of manure-based urea pellets on growth, yield, and nitrate content in coriander, garden cress, and parsley plants	pot	-	
244	2018	Avidov	Using polyethylene sleeves with forced aeration for composting olive mill wastewater pre-absorbed by vegetative waste	Petri dish	N/A	Saadi *et al.*, 2013
245	2018	Benavente	Choice of pyrolysis parameters for urban wastes affects soil enzymes and plant germination in a Mediterranean soil	Petri dish	N/A	
246	2018	Bonanomi	Comparing chemistry and bioactivity of burned vs. decomposed plant litter: different pathways but same result?	Petri dish	N/A	Phytotoxkit™(MicroBioTests Inc.) in accordance with the manufacturer’s instruction (http://www.microbiotests.be/)
247	2018	Finngean	Plant assays and avoidance tests with collembola and earthworms demonstrate rehabilitation success in bauxite residue	N/A	software "Image Tools"	

248	2018	Hagner	Assessing toxicity of metal contaminated soil from glassworks sites with a battery of biotests	Petri dish	-	OECD, 2006
249	2018	Khan	Uptake and phytotoxic effect of benzalkonium chlorides in *Lepidium sativum* and *Lactuca sativa*	Petri dish	N/A	
250	2018	Konczak	Application of biochar to sewage sludge reduces toxicity and improve organisms’ growth in sewage sludge-amended soil in long term field experiment	Petri dish	software "Image Tool 3.0"	Phytotoxkit F; ISO, 2016. Phytotoxkit: ISO 18763:2016; OECD, 1984
251	2018	Lawrence	Construction of fluorescent analogs to follow the uptake	N/A	-	
252	2018	Marchand	Pilot scale aided-phytoremediation of a co-contaminated soil	plastic pots	-	ISO 11269-2 (ISO, 1995)
253	2018	Marra	Biochars from olive mill waste have contrasting effects on plants, fungi and phytoparasitic nematodes	Petri dish	N/A	
254	2018	Masi	Lentiquinones A, B, and C, phytotoxic anthraquinone derivatives isolated from *Ascochyta lentis*, a pathogen of lentil	Petri dish	N/A	
255	2018	Oleszczuk	Changes of total and freely dissolved polycyclic aromatic hydrocarbons and toxicity of biochars treated with various aging processes	test plates	software "Image Tool 3.0"	Phytotoxkit, Phytotestkit; MicroBioTests Inc. (Gent, Belgium)
256	2018	Palas	Catalytic wet air oxidation of Reactive Black 5 in the presence of $LaNiO_3$ perovskite catalyst as a green process for azo dye removal	Petri dish	N/A	Almeida and Corso, 2014; Hoekstra *et al.,* 2002

257	2018	Papadaki	Changes in phenolic compounds and phytotoxicity of the spanish style green olive processing wastewaters by *Aspergillus niger* B60	Petri dish	N/A	
258	2018	Reichl	Uptake and metabolism of the antidepressants sertraline, clomipramine, and trazodone in a garden cress (*Lepidium sativum*) model	Petri dish	-	
259	2018	Rybczynska-Tkaczyk	Comparative study of eco- and cytotoxicity during biotransformation of anthraquinone dye Alizarin Blue Black B in optimized cultures of microscopic fungi	N/A	N/A	Rybczyńska-Tkaczyk and Korniłłowicz-Kowalska, 2017
260	2018	Tarnawski	Use of chemical indicators and bioassays in bottom sediment ecological risk assessment	N/A	N/A	Phytotoxkit, 2004; Baran and Tarnawski, 2013 & 2015
261	2018	Tigini	Tannerymixed liquors froman ecotoxicological and mycological point of view: Risks vs potential biodegradation application	N/A	N/A	UNICHIM No. 1651, 2003
262	2018	Tzvetkova	Effect of herbicides paraquat and glyphosate on the early development of two tested plants	Petri dish	N/A	Lyubenova 2000
263	2018	Yokawa	Anaesthetics stop diverse plant organ movements, affect endocytic vesicle recycling and ROS homeostasis, and block action potentials in Venus flytraps	Petri dish	-	
264	2019	Okada	Phytotoxic activity of kiwifruit leaves and isolation of a phytotoxic substance	Petri dish	N/A	
265	2019	Sakamoto	Evaluation of allelopathic competency of *Lamium amplexicaule* and identification of its allelopathic active substance	Petri dish	N/A	

266	2019	Akabli	Molecular docking, ADME/Tox prediction, and *in vitro* study of the cell growth inhibitory activity of five β-carboline alkaloids	Petri dish	N/A	
267	2019	Balestri	Phytotoxicity assessment of conventional and biodegradable plastic bags using seed germination test	Petri dish	software "ImageJ2" (Rueden *et al.,* 2017)	
268	2019	Demasi	Ailanthone from *Ailanthus altissima* (Mill) Swingle as potential natural herbicide	Petri dish	N/A	ISTA, 2011 (International Seed Testing Association)
269	2019	Galli	Ecotoxicity of foaming agent conditioned soils tested on two terrestrial organisms	Petri dish	N/A	US EPA, 1996
270	2019	Golkar	Production of a new mucilage compound in *Lepidium sativum* callus by optimizing *in vitro* growth conditions	N/A	-	
271	2019	Hadian	Evaluation of *in vitro* mucilage and lepidine biosynthesis in different genotypes of *Lepidium sativum* Linn originated from Iran	on semi-solid MS medium	-	
272	2019	Haghighi	Ontogenetic variability of *Vitex pseudo-negundo* essential oil and its phytotoxic activity	Petri dish	N/A	Saharkhiz *et al.*, 2010
273	2019	Intani	Phytotoxicity of corncob biochar before and after heat treatment and washing	tray	digital slide caliper	
274	2019	Khezrianjoo	Photodestruction of Direct Yellow 11 in aqueous TiO_2 suspension: effect of operational parameters on detoxification, Langmuir-Hinshelwood kinetic expression and biodegradability	Petri dish	N/A	

275	2019	Matousková	Phytotoxic effect of invasive *Heracleum mantegazzianum* essential oil on dicot and monocot species	Petri dish	N/A	
276	2019	Mierzejewska	Removal and ecotoxicity of 2,4-D and MCPA in microbial cultures enriched with structurally-similar plant secondary metabolites	N/A	-	Phytotoxkit, MicroBioTests Inc. https://www.microbiotests.com/SOPs/Phytotestkit%20(complete%20test)%20SOP%20-%20A5.pdf
277	2019	Mierzwa-Hersztek	Assessment of energy parameters of biomass and biochars, leachability of heavy metals and phytotoxicity of their ashes	Petri dish	N/A	
278	2019	Perveen	Bioherbicidal potential of some allelopathic agroforestry and fruit plant species against *Lepidium sativum*	Petri dish	N/A	
279	2019	Pii	Phytotoxicity alleviation by bacterial species isolated from polycyclic aromatic hydrocarbons (PAHs) contaminated sites	Petri dish	software WinRhizo	
280	2019	Schrey	Aminotenuazonic acid: isolation, structure elucidation, total synthesis and herbicidal activity of a new tetramic acid from fruiting bodies of *Laccaria* species	test tubes	N/A	Anke *et al.*, 1989
281	2019	Tamás	Steps in organic fraction of municipal solid waste composting and compost quality evaluation	Petri dish	-	
282	2019	Tatusko-Krygier	Application of biological methods to assess the toxicity of soils contaminated with heavy metals and the effectiveness of stabilisation processes	Petri dish	software "ImageJ 1.8.0"	

283	2019	Urbaniak	The application of different biological remediation strategies to PCDDs/PCDFs contaminated urban sediments	N/A	N/A	Phytotoxkit™ ; Phytotoxkit 2004
284	2019	Víglas	*Trichoderma atroviride*: an isolate from forest environment with secondary metabolites with high antimicrobial potential	Petri dish	N/A	
285	2019	Ajdanian	The growth and development of cress (*Lepidium sativum*) affected by blue and red light	plastic pots	tape measure with 0.01 m precision	
286	2019	Bessa	Carbamazepine is degraded by the bacterial strain *Labrys portucalensis* F11	Petri dish	N/A	OECD, 2006
287	2019	Bosker	Microplastics accumulate on pores in seed capsule and delay germination and root growth of the terrestrial vascular plant Lepidium sativum	Petri dish	measured manually using a ruler under a dissection microscope	Hoekstra *et al.*, 2002)
288	2019	Cristina	Evaluation of anaerobic digestates from sewage sludge as a potential solution for improvement of soil fertility	Petri dish	N/A	DIVAPRA *et al.,* 1998
289	2019	Della Pepa	Antimicrobial and phytotoxic activity of *Origanum heracleoticum* and *O. majorana* essential oils growing in Cilento (Southern Italy)	Petri dish	N/A	
290	2019	Demasi	Ailanthone inhibition data on seed germination and seedling growth of *Lepidium sativum* L. and *Raphanus sativus* L.	plastic flasks	N/A	
291	2019	Doesburg	Empirical investigation of preparations produced according to the European Pharmacopoeia monograph 1038	plastic bags	-	Baumgartner and Flückiger, 2001; Baumgartner *et al.*, 2012

292	2019	Emhofer	High-performance liquid chromatography drift-tube ion-mobilityquadrupole time-of-flight/mass spectrometry for the identity confirmation and characterization of metabolites from three statins (lipid-lowering drugs) in the model plant cress (*Lepidium sativum*) after uptake from water	rectangular dish	-	
293	2019	Garbo	Assessment of the ecotoxicity of phytotreatment substrate soil as landfill cover material for in-situ leachate management	Petri dish	N/A	
294	2019	Gmurek	Comparison of radical-driven technologies applied for paraben mixture degradation: mechanism, biodegradability, toxicity and cost assessment	N/A	N/A	Gomes *et al.,* 2017; Wang, 1992
295	2019	Golkar	Production of a new mucilage compound in *Lepidium sativum* callus by optimizing *in vitro* growth conditions	N/A	-	
296	2019	Gomes	Analysis of potentially toxic metal constraints to apply sewage sludge in Portuguese agricultural soils	Petri dish	N/A	
297	2019	Hijazin	Uptake and modification of umbelliferone by various seedlings	Petri dish	-	
298	2019	Ida	Tree fern *Cyathea lepifera* may survive by its phytotoxic property	Petri dish	N/A	
299	2019	Jani	Physicochemical and toxicological characterization of hazardous wastes from an old glasswork dump at southeastern part of Sweden	plastic pots	N/A	

300	2019	Joniec	Effect of reclamation treatments on microbial activity and phytotoxicity of soil degraded by the sulphur mining industry	Petri dish	-	
301	2019	Ju	Exploring the chelation-based plant strategy for iron oxide nanoparticle uptake in garden cress (*Lepidium sativum*) using magnetic particle spectrometry	Petri dish	-	
302	2019	Kebibeche	Addition of wood sawdust during the co-composting of sewage sludge and wheat straw influences seeds germination	Petri dish	N/A	
303	2019	Krumsri	Evaluation of the allelopathic potential of leaf extracts from *Dischidia imbricata* (Blume) steud	Petri dish	N/A	
304	2019	Kun	Glucopyranosylidene-spiro-benzo[b][1,4]oxazinones and -benzo[b][1,4]thiazinones: Synthesis and investigation of their effects on glycogen phosphorylase and plant growth inhibition	Petri dish	N/A	
305	2019	Maiorana	Phytotoxicity of wear debris from traditional and innovative brake pads	Petri dish	N/A	Martignon, 2009; Baudo, 2013
306	2019	Matouskova	Phytotoxic effect of invasive *Heracleum mantegazzianum* essential oil on dicot and monocot species	Petri dish	N/A	
307	2019	Nikolaeva	Ecotoxicological effects of traffic-related pollutants in roadside soils of Moscow	Petri dish	manually with a ruler	
308	2019	Oleszczuk	Impact of ZnO and ZnS nanoparticles in sewage sludge-amended soil on bacteria, plant and invertebrates	glass jars	software "Image Tool 3.0"	Phytotoxkit F test; Collembola test

309	2019	Patz	Culture dependent and independent analysis of potential probiotic bacterial genera and species present in the phyllosphere of raw eaten produce	growth trays	-	
310	2019	Ravindran	Influence of biochar on physico-chemical and microbial community during swine manure composting process	N/A	N/A	Zucconi *et al.*, 1981; Wang *et al., 2016*
311	2019	Schrey	Aminotenuazonic acid: Isolation, structure elucidation, total synthesis and herbicidal activity of a new tetramic acid from fruiting bodies of *Laccaria species*	test tubes	N/A	Anke *et al.*, 1989
312	2019	Schrey	E- and Z-Proxamidines, unprecedented 1,3-Diazacyclooct-1-ene alkaloids from fruiting bodies of *Laccaria proxima*	test tubes	N/A	Anke *et al.*, 1990
313	2019	Smeriglio	Characterization and phytotoxicity assessment of essential oils from plant byproducts	Petri dish	N/A	
314	2019	Sousa	Hazardous impact of vinasse from distilled winemaking by-products in terrestrial plants and aquatic organisms	glass Petri dish	N/A	OECD, 2006; USEPA, 1996 & 2012
315	2019	Thligene	Effect of coffee silver skin and brewers' spent grain in the control of root-knot nematodes	N/A	N/A	Zucconi *et al.*, 1981
316	2019	Ullah	Effect of ultraviolet-C radiation and melatonin stress on biosynthesis of antioxidant and antidiabetic metabolites produced in *in vitro* callus cultures of *Lepidium sativum* L.	plates	N/A	Lagrimini, 1980
317	2020	Kyaw	Allelopathic potential of *Acacia pennata Willd* leaf extracts against the seedling growth of six test plants	Petri dish	N/A	

318	2020	Ajdanian	Investigation of photosynthetic effects, carbohydrate and starch content in cress (*Lepidium sativum*) under the influence of blue and red spectrum	pot	-	
319	2020	Al dayel	Investigating the applications of *Chlorella vulgaris* in agriculture and nanosilver production	on cotton wool	N/A	
320	2020	Alghonmeen	Assessment of exogenous application of plant growth regulators on cress seed germination and β-galactosidase activity	Petri dish	-	
321	2020	Bargougui	Co-composting of olive industry wastes with poultry manure and evaluation of the obtained compost maturity	N/A	N/A	Zucconi *et al*., 1981
322	2020	Barroso	Fire effects on soils - a pilot scale study on the soils affected by wildfires in the Czech republic	plate	N/A	Phytotoxkit™ ; Phytotoxkit 2004
323	2020	Fernandes	N-doped titanium dioxide for mixture of parabens degradation based on ozone action and toxicity evaluation: Precursor of nitrogen and titanium effect	plate	N/A	Zucconi *et al.,* 1981 with changes proposed by Komilis *et al*., 2005; following international guidelines and criteria (USEPA,1996, 2012)
324	2020	Jarosz-Krzeminska	Repurposing fly ash derived from biomass combustion in fluidized bed boilers in large energy power plants as a mineral soil amendment	plate	N/A	
325	2020	Khaled	Phytotoxic effect of macerates and mulches from *Cupressus leylandii* leaves on clover and cress: role of chemical composition	Petri dish	-	
326	2020	Masum	Assessment of the allelopathic potential and identification of the phytotoxic substances from the straw of Bangladeshi indigenous rice variety "goria"	Petri dish	N/A	

327	2020	Matinkhah	Nitrogen-fixing potential of *Halimodendron halodendron* (Pall.) Voss in arid and semi-arid areas of Iran	plastic pot	-	
328	2020	Mehta	Assessment of the possible reuse of extractive waste coming from abandoned mine sites: case study in Gorno, Italy	more details in supplement	N/A	Wundram *et al.,* 1997; Blok *et al.,* 2008
329	2020	Mohamed	Effects of organic and inorganic fertilization on growth, yield, seed fixed oil content, and fatty acids profile of garden cress (*Lepidium sativum* L.)	field	-	
330	2020	Morau	Interactions between abiotic factors and the bioactivity of biodynamic horn manure on the growth of garden cress (*Lepidium sativum* L) in a bioassay	bags	software "Sigma Scan Pro 5.0"	Baumgartner *et al.,* 2013
331	2020	Morau	Growth responses of garden cress (*Lepidium sativum* L.) to biodynamic cow manure preparation in a bioassay	bags	software "Sigma Scan Pro 5.0"	Baumgartner *et al.,* 2014
332	2020	Neubauer	Performance of the kraft mill biosolids compost and their quality evaluation	Petri dish	N/A	
333	2020	Ogórek	Plant-fungal interactions: a case study of *Epicoccoum nigrum* link	Petri dish	digital caliper	
334	2020	Peduto	Phytotoxicological assessment and its relationship with environmental variables of Rio Grande Reservoir waters (Sao Paulo State, Brazil)	Petri dish	digital caliper	
335	2020	Perboni	Herbicidal activity of kidney leaf mud plantain leaves extracts on the germination of four species	Petri dish	ruler	

336	2020	Radlinska	Assessment of the "Olawa" smelter (Olawa, Southwest Poland) on the environment with ecotoxicological tests	N/A	N/A	Phytotoxkit F test in assessment of toxicity of two types of sewage sludge standard operational procedure (2017)
337	2020	Smolinska	The influence of compost and nitrilotriacetic acid on mercury phytoextraction by *Lepidium sativum* L	pot	-	
338	2020	Sobik-Szoltysek	Toxicity evaluation of eluates from waste after thermal conversion of sewage sludge	Petri dish	N/A	Walter *et al.*, 2006
339	2020	Stanczyk-Mazanek	Evaluation of the effect of toxicity of biochar used for soil fertilization and its water extract on plants	Petri dish	N/A	
340	2020	Tarkowska	Plant triterpenoid crosstalk: the interaction of brassinosteroids and phytoecdysteroids in *Lepidium sativum*	vertical plate	software "ImageJ"	
341	2020	Bajwa	Toxic potential and metabolic profiling of two australian biotypes of the invasive plant parthenium weed (*Parthenium hysterophorus* L.)	pots	N/A	
342	2020	Bandarra	Assessment of hazardous property HP 14 using ecotoxicological tests: a case study of weathered coal fly ash	Petri dish	N/A	
343	2020	Bonanomi	Decomposition and organic amendments chemistry explain contrasting effects on plant growth promotion and suppression of *Rhizoctonia solani* damping off	Petri dish	N/A	
344	2020	Bozym	Assessment of phytotoxicity of leachates from landfilled waste and dust from foundry	Petri dish	N/A	Zucconi *et al.*, 1981

345	2020	Brzeszcz	Hydrocarbon removal by two differently developed microbial inoculants and comparing their actions with biostimulation treatment	N/A	N/A	PhytotoxkitTM test (MicroBioTests Inc., Nazareth, Belgium)
346	2020	Chemetova	Towards sustainable valorisation of *Acacia melanoxylon* biomass: Characterization of mature and juvenile plant tissues	Petri dish	N/A	(CEN, 2011), (Chemetova et al., 2018)
347	2020	Dehmani	Chemical characterization and adsorption of oil mill wastewater on Moroccan clay in order to be used in the agricultural field	Petri dish	N/A	
348	2020	Di Carlo	Ecotoxicological risk assessment of revegetated bauxite residue: Implications for future rehabilitation programmes	test plates	software "ImageJ"	Phytotoxkit™ (MicroBioTests Inc.)
349	2020	Diaconu	Characterization of heavy metal toxicity in some plants and microorganisms - A preliminary approach for environmental bioremediation	Petri dish	N/A	
350	2020	Dobrucka	Phytotoxic effects of biosynthesized ZnO nanoparticles using *Betonica officinalis* extract	Petri dish	N/A	
351	2020	Elshafie	Biological and spectroscopic investigations of new tenoxicam and 1.10-phenthroline metal complexes	Petri dish	N/A	
352	2020	Fernandes	Photocatalytic ozonation of parabens mixture using 10% N-TiO_2 and the effect of water matrix	Petri dish	N/A	
353	2020	Fernandes	Fungal biodegradation and multi-level toxicity assessment of vinasse from distillation of winemaking by-products	Petri dish	N/A	Zucconi *et al.*, 1981 with changes introduced by Komilis *et al.*, 2005; USEPA, 1996, 2012

354	2020	Gomes	Thermal dehydration of urban biosolids with green liquor dregs from pulp and paper mill	Petri dish	N/A	
355	2020	Grul'ová	Thymol chemotype *Origanum vulgare* L. essential oil as a potential selective bio-based herbicide on monocot plant species	Petri dish	N/A	
356	2020	Konczak	Carbon dioxide as a carrier gas and mixed feedstock pyrolysis decreased toxicity of sewage sludge biochar	Petri dish	software "Image Tool 3.0"	Phytotoxkit F; ISO, 2016. Phytotoxkit: ISO 18763:2016; OECD, 1985
357	2020	MacDonald	An oxygen delivery polymer enhances seed germination in a martian-like environment	planter containers	N/A	
358	2020	Mierzejewska	Biodegradation potential and ecotoxicity assessment in soil extracts amended with phenoxy acid herbicide (2,4-D) and a structurally similar plant secondary tabolite (ferulic acid)	N/A	N/A	Phytotoxkit, 2004; Baran and Tarnawski 2013; Urbaniak *et al*., 2016; Mierzejewska *et al*., 2017
359	2020	Pflugmacher	The influence of new and artificial aged microplastic and leachates on the germination of *Lepidium sativum* L.	glass dish	N/A	
360	2020	Pignattelli	Physiological responses of garden cress (*L. sativum*) to different types of microplastics	test plate, pots	N/A	Phytotoxkit (MicroBioTests Inc.)
361	2020	Rollinson	Anaerobic digestion of mercury phytoextraction crops with intermediary stage bio-waste polymer treatment	bioreactors	-	
362	2020	Rybczynska-Tkaczyk	Biotransformation and toxicity effect of monoanthraquinone dyes during *Bjerkandera adusta* CCBAS 930 cultures	N/A	N/A	Rybczyńska-Tkaczyk and Korniłłowicz-Kowalska, 2018

363	2020	Sattari Vayghan	Growth temperature influence on lipids and photosynthesis in *Lepidium sativum*	pots	length: manually, leaf area: software "ImageJ"	
364	2020	Schiavo	Adverse effects of oxo-degradable plastic leachates in freshwater environment	Petri dish	N/A	
365	2020	Spina	Ecofriendly laccases treatment to challenge emerging contaminants issue	N/A	-	UNICHIM No. 1651, 2003
366	2020	Steliga	Application of *Festuca arundinacea* in phytoremediation of soils contaminated with Pb, Ni, Cd and petroleum hydrocarbons	N/A	N/A	Phytotoxkit™; Baran and Tarnawski, 2013; Blinova *et al.*, 2012; Maminidy-Pajany *et al.*, 2011
367	2020	Szara	Ecotoxicological characteristics and ecological risk assessment of trace elements in the bottom sediments of the Rożnów reservoir (Poland)	test plates	software "ImageJ"	Phytotoxkit, 2004
368	2020	Tomczyk	Polycyclic aromatic hydrocarbons (PAHs) persistence, bioavailability and toxicity in sewage sludge- or sewage sludge-derived biochar-amended soil	Petri dish	software "Image Tool 3.0"	Phytotestkit, Phytotoxkit F test (MicroBioTests Inc., Nazareth, Belgium)
369	2020	Urbaniak	Evaluation of ecotoxicological and chemical properties of soil amended with Hudson River (New York, USA) sediment	N/A	N/A	Phytotoxkit, 2004; commercial toxicity bioassay kit (Environmental Bio-Detection Product Inc., Mississauga, Ontario, Canada)
370	2020	Urbaniak	Effects of soil amendment with PCB-contaminated sediment on the growth of two cucurbit species	N/A	N/A	Phytotoxit, 2004; Baran and Tarnawski, 2013; Antonkiewicz *et al.*, 2018; Kopeć *et al.*, 2013;

						Mierzwa-Hersztek *et al.*, 2017; Mierzejewska *et al.*, 2018
371	2020	Volta	Ecotoxicological effects of atmospheric particulate produced by braking systems on aquatic and edaphic organisms	Petri dish	N/A	UNICHIM No. 1651, 2003
372	2020	Wrobel	Magnesium–isotope fractionation in chlorophyll-a extracted from two plants with different pathways of carbon fixation (C3, C4)	trays	-	
373	2020	Falanga	Ecotoxicity evaluation of pristine and indolicidin-coated silver nanoparticles in aquatic and terrestrial ecosystem	Petri dish	N/A	
374	2020	Rob	Potential use of *Schumannianthus dichotomus* waste: the phytotoxic activity of the waste and its identified compounds	Petri dish	N/A	
375	2021	Islam	Isolation and identification of three potential phytotoxic compounds from *Chrysopogon aciculatus*	Petri dish	N/A	
376	2021	Krumsri	Isolation and Identification of Two Potent Phytotoxic Substances from *Afzelia xylocarpa* for Controlling Weeds	Petri dish	N/A	
377	2021	Aldayel	Isolation and characterization of bacteria from tomato and assessment of its plant growth-promoting traits in three economically important crops in Al-Ahsa region, Saudi Arabia	plastic pot	N/A	
378	2021	Alotaibi	*Lepidium sativum* sprouts grown under elevated CO_2 hyperaccumulate glucosinolates and antioxidants	tray	N/A	

			and exhibit enhanced biological and reduced antinutritional properties			
379	2021	Barroso	Assessing the ecotoxicity of soil affected by wildfire	plate	N/A	Phytotoxkit™; Phytotoxkit 2014
380	2021	Carella	Research Paper Thermal conversion of fish bones into fertilizers and biostimulants for plant growth-A low tech valorization process for the development of circular economy in least developed countries	Petri dish	N/A	
381	2021	De Martino	Variations in composition and bioactivity of *Ocimum basilicum* cv 'Aroma 2' essential oils	Petri dish	N/A	
382	2021	Demasi	Functionalized dextrin-based nanosponges as effective carriers for the herbicide ailanthone	plastic flasks	N/A	ISTA, 2011 (International Seed Testing Association)
383	2021	Esmaeili	Comparison of coating and nano-coating of chitosan-*Lepidium sativum* seed gum composites on quality and shelf life of beef	water bath	-	
384	2021	Golkar	The effects of nanographene oxide on the morpho-biochemical traits and antioxidant activity of *Lepidium sativum* L. under *in vitro* salinity stress	on solidified MS basal medium	-	
385	2021	Haghighi	Phytotoxic potential of *Vitexpseudo-negundo* leaf and flower extracts and analysis of phenolic compounds	plate	-	
386	2021	Kepys	Assessment of ecotoxicity of incinerated sewage sludge ash (ISSA)	Petri dish	N/A	
387	2021	Keutgen	Nutritional and sensory quality of two types of cress microgreens depending on the mineral nutrition	trays of the Cressbar® company		

388	2021	Lincho	TiO_2 nanotube catalysts for parabens mixture degradation by photocatalysis and ozone-based technologies	Petri dish	N/A	
389	2021	Malafatti	Prozac® removal promoted by HAP:Nb_2O_5 nanoparticles system: by-products, mechanism, and cytotoxicity assessment	filter paper	N/A	Malas *et al*., 2018
390	2021	Merad	Essential oils from two apiaceae species as potential agents in organic crops protection	Petri dish	N/A	2021
391	2021	Michalak	Effect of *Fucus* extract and biomass enriched with Cu(II) and Zn(II) ions on the growth of garden cress (*Lepidium sativum*) under laboratory conditions	Petri dish	N/A	
392	2021	Nouri	Improving seed germination and seedling growth of *Lepidium sativum* with different priming methods under arsenic stress	Petri dish	N/A	
393	2021	Perveen	Allelopathic hormesis and potent allelochemicals from multipurpose tree *Moringa oleifera* leaf extract	Petri dish	N/A	
394	2021	Qasem	Establishment, growth and productivity of selected medicinal plant species in the arid zone 100-200mm rainfall in Jordan	field	N/A	
395	2021	Seifarth	Duration and timing interactions of early-life stress and the potential for recovery	transparent container	N/A	
396	2021	Sourková	Phytotoxicity of tires evaluated in simulated conditions	test kit	software "Image Tool 3.0"	Phytotoxkit™ ; Phytotoxkit 2004; https://www.microbiotests.com/wp-content/uploads/2019/05/Phytotoxicity-test_Phytotoxkit-

						solidsamples_Standard-Operating-Procedure.pdf
397	2021	Szymanski	Evaluation of phytotoxicity of bimetallic Ag/Au nanoparticles synthesized using *Geum urbanum* L.	on substrate	N/A	
398	2021	Tsekhmister	Plant growth regulatory activity in the phytopathogenic fungus *Plectosphaerella melonis* strain 502	Petri dish	N/A	
399	2021	Vannucchi	De-inked paper sludge and mature compost as high-value components of soilless substrate to support tree growth	Petri dish	N/A	
400	2021	Zagula	Preliminary research on the influence of a pulsed magnetic field on the cationic profile of sunflower, cress, and radish sprouts and on their germination rate	Petri dish	N/A	
401	2021	Buzmakov	Ecological criteria for assessing the content of petroleum hydrocarbons in the main soils of coniferous - deciduous forests and forest steppe	container	N/A	
402	2021	Gigante	On the use of paper sludge as filler in biocomposites for injection moulding	Petri dish	N/A	
403	2021	Hamdan	Biological treatment of hazardous heavy metals by *Streptomyces rochei* ANH for sustainable water management in agriculture	Petri dish	N/A	
404	2021	Hossen	Three active phytotoxic compounds from the leaves of *Albizia richardiana* (voigt.) King and prain for the development of bioherbicides to control weeds	Petri dish	N/A	

405	2021	Mousavi	Phytotoxic potential and phenolic profile of extracts from *Scrophularia striata*	Petri dish	ruler	
406	2021	Naddafi	Chlorpyrifos remediation in agriculture runoff with homogeneous solar photo-Fenton reaction at near neutral pH: phytotoxicity assessment	Petri dish	N/A	
407	2021	Nikolaeva	Linking pollution of roadside soils and ecotoxicological responses of five higher plants	Petri dish	ruler	
408	2021	Mohamed	Biochemical characterization, phytotoxic effect and antimicrobial activity against some phytopathogens of new gemifloxacin schiff base metal complexes	Petri dish	N/A	
409	2021	Montvydienė	Toxicological effects of different-sized Co–Fe ($CoFe_2O_4$) nanoparticles on *Lepidium sativum* L.: towards better understanding of nanophytotoxicity	Petri dish	N/A	
410	2021	Pflugmacher	Ageing affects microplastic toxicity over time: Effects of aged polycarbonate on germination, growth, and oxidative stress of *Lepidium sativum*	glass dish	digital caliper	
411	2021	Photiou	Recovery of phosphate from dewatered anaerobic sludge and wastewater by thermally treated *P. oceanica* residues and its potential application as a fertilizer	Petri dish	N/A	
412	2021	Pignatelli	Short-term physiological and biometrical responses of *Lepidium sativum* seedlings exposed to PET-made microplastics and acid rain	test plate	precision caliper	
413	2021	Pignatelli	Effects of polyethylene terephthalate (PET) microplastics and acid rain on physiology and growth of *Lepidium sativum*	N/A	precision caliper	Pignattelli *et al.*, 2021

414	2021	Siciliano	Cerium, gadolinium, lanthanum, and neodymium effects in simplified acid mine discharges to *Raphidocelis subcapitata*, *Lepidium sativum*, and *Vicia faba*	Petri dish	N/A	Libralato *et al.*, 2016
415	2021	Šrédlová	The sensitivity of multiple ecotoxicological assays for evaluating *Microcystis aeruginosa* cellular algal organic matter and contribution of cyanotoxins to the toxicity	Petri dish	N/A	
416	2021	Steliga	Assessment of the suitability of *Melilotus officinalis* for phytoremediation of soil contaminated with petroleum hydrocarbons (TPH and PAH), Zn, Pb and Cd based on toxicological tests	polystyrene test plates	N/A	Phytotoxkit™
417	2021	Tomczyk	Ecotoxicological assessment of sewage sludge-derived biochars-amended soil	Petri dish	software "Image Tool 3.0"	Phytotoxkit test
418	2021	Wieckol-Ryk	Solid peroxy compounds as additives to organic waste for reclamation of post-industrial contaminated soils	Petri dish	N/A	
419	2021	Yildirim	Humic + Fulvic acid mitigated Cd adverse effects on plant growth, physiology and biochemical properties of garden cress	polyethylene pots	N/A	
420	2021	Celetti	Phytotoxicity of hydrochars obtained by hydrothermal carbonization of manure-based digestate	Petri dish	software "WinRHIZO"	
421	2021	Chung	Effect of biochar amendment on compost quality, gaseous emissions and pathogen reduction during in-vessel composting of chicken manure	N/A	-	Zucconi *et al.*, 1981

422	2021	Methneni	Persistent organic and inorganic pollutants in the effluents from the textile dyeing industries: Ecotoxicology appraisal via a battery of biotests	Petri dish	N/A
423	2021	Serino	Biodegradable polymers as carriers for tuning the release and improve the herbicidal effectiveness of *Dittrichia viscosa* plant organic extracts	Petri dish	-
424	2022	Khatun	Allelopathic activity of *Annona reticulata* L. leaf extracts and identification of three allelopathic compounds	Petri dish	N/A
425	2022	Krumsri	Phytotoxic effects of *Senna garrettiana* and identification of phytotoxic substances for the development of bioherbicides	Petri dish	N/A
426	2022	Kyaw	Assessment of the phytotoxic potential of *Dregea volubilis* benth. Ex hook.f. and identification of its phytotoxic substances for weed control	Petri dish	N/A
427	2022	Lun	Two allelopathic substances from *Plumbago rosea* stem extracts and their allelopathic effects	Petri dish	ruler
428	2022	Matuda	Allelopathy and allelopathic substances of fossil tree species *Metasequoia glyptostroboides*	Petri dish	ruler
429	2022	Moh	Allelopathic activity of a novel compound, 5,6-dihydrogen-11α-tigloyl-17β-marsdenin, and a known steroidal glycoside from the leaves of *Marsdeniatenacissima tenacissima* (roxb.) Moon	Petri dish	N/A
430	2022	Aminifard	Changes in biochemical and morphological characters of garden cress (*Lepidium sativum* L.) as	Petri dish	ruler

			affected by foliar application with casein amino acid and salicylic acid under greenhouse conditions			
431	2022	Babaei	Improving the effects of salt stress by β-carotene and gallic acid using increasing antioxidant activity and regulating ion uptake in *Lepidium sativum* L.	Petri dish	-	
432	2022	Bakhtiari	The effects of callus elicitation on lepidine, phenolic content, and antioxidant activity of *Lepidium sativum* L.: chitosan and gibberellic acid	N/A	-	
433	2022	Boujelben	Box-Behnken approach for optimization of Cr(III) removal from a real tanning effluent using powdered marble	on solidified MS basal medium	-	Zucconi *et al*., 1981
434	2022	Fragalà	New insights into municipal biowaste derived products as promoters of seed germination and potential antifungal compounds for sustainable agriculture	Petri dish	digital caliper	
435	2022	Gautam	Effect of different organic manures on production of *Lepidium sativum* under morus based agroforestry system	field	with help of scale	
436	2022	Lugovitskaya	Sulfite lignin nanoparticles and nanovesicles as biologically active crop growth stimulants	Petri dish	N/A	
437	2022	Nassar	Effect of a veterinary antibiotic on the growth of regularly consumed Lebanese plants	Petri dish	N/A	Tiquia *et al*., 1996
438	2022	Photiou	Calcined eggshells in anaerobic digestion: Buffering acidification in AD and evaluating end products from phosphate adsorption as soil conditioners	Petri dish	N/A	

439	2022	Sedefoglu	Green synthesized ZnO nanoparticles using *Ganoderma lucidum*: characterization and *in vitro* nanofertilizer effects	Petri dish	N/A	
440	2022	Sliwka	Analysis of the properties of coal sludge in the context of the possibility of using it in biological reclamation	Petri dish	N/A	
441	2022	Soriano	Iridoid glycosides isolated from B*ellardia trixago* identified as inhibitors of *Orobanche cumana* radicle growth	Petri dish	stereoscopic microscope (Leica)	
442	2022	Szymanski	Application of phytotests to study of environmental safety of biologicaly synthetised au and au/zno nanoparticles using *Tanacetum parthenium* extract	in incubator	N/A	
443	2022	Türkyilmaz	A comparative study of free chlorine activated by Fe+2 and UV C light catalysts in the treatment of real and simulated textile wastewater: Optimization, reactive species and phytotoxicity assessment	Petri dish	N/A	
444	2022	Vaz	Evaluation of the activation procedure on oxone efficiency for synthetic olive mill wastewater treatment	Petri dish	N/A	Trautmann and Krasny, 2017
445	2022	Avona	Preliminary insights about the treatment of contaminated marine sediments by means of bioslurry reactor: Process evaluation and microbiological characterization	Petri dish	N/A	
446	2022	Boudali	Zincum Metallicum, a homeopathic drug, alleviates Zn-induced toxic effects and promotes plant growth and antioxidant capacity in *Lepidium sativum* L	Petri dish, plant pot	N/A	

447	2022	Godlewska	Ecotoxicity of sewage sludge- or sewage sludge/willow-derived biochar-amended soil	N/A	N/A	Phytotoxkit F Test; ISO guideline 18763 (ISO, 2016)
448	2022	Godlewska	Effect of carrier gas change during sewage sludge or sewage sludge and willow pyrolysis on ecotoxicity of biochar-amended soil	Petri dish	software "Image Tool 3.0"	Phytotoxkit F Test; ISO guideline 18763 (ISO, 2016)
449	2022	Lachkar	*In vitro* antimitotic and hypoglycemic effect study and acute toxicity assessment of the aqueous and organic extracts of *Chamaerops humilis* L. var. argentea Andre	Petri dish	N/A	Gagiu *et al*., 1973
450	2022	Liwarska-Bizukojc	Phytotoxicity assessment of biodegradable and non-biodegradable plastics using seed germination and early growth tests	Test plate	image analysis software, i. e. NIS ELEMENTS AR software (Nikon, Japan)	Phytotoxkit
451	2022	Liwarska-Bizukojc	Application of a small scale-terrestrial model ecosystem (STME) for assessment of ecotoxicity of bio-based plastics	microcosm	image analysis software, i. e. NIS ELEMENTS AR software (Nikon, Japan)	
452	2022	Miranda	Can aged microplastics be transport vectors for organic micropollutants? - Sorption and phytotoxicity tests	test plate	N/A	Phytotoxkit for liquid samples (Microbiotests Inc., 2016) in accordance with ISO Standard 18763 (2016)
453	2022	Passatore	Morpho-physiological and molecular responses of *Lepidium sativum* L. seeds induced by bismuth exposure	Petri plate	software "ImageJ"	

454	2022	Santini	Un-biodegradable and biodegradable plastic sheets modify the soil properties after six months since their applications	Petri dish	N/A	
455	2022	Villani	Inuloxin a inhibits seedling growth and affects redox system of Lycopersicon *esculentum* mill. And *Lepidium sativum* L..	Petri dish	N/A	Moeini *et al.*, 2019
456	2022	Babaei	Improving the effects of salt stress by β-carotene and gallic acid using increasing antioxidant activity and regulating ion uptake in *Lepidium sativum* L.	Petri dish	-	
457	2022	Bożym	Assessment of phytotoxicity of landfilled waste and foundry dust based on the direct test	Petri dish	-	
458	2022	Golpe	Chlorhexidine residues in sludge from municipal wastewater treatment plants: analytical determination and toxicity evaluation	plates	-	
459	2022	Nakabayashi	The phytotoxin myrigalone a triggers a phased detoxification programme and inhibits *Lepidium sativum* seed germination via multiple mechanisms including interference with auxin homeostasis	Petri dish	-	
460	2022	Vasilyeva	Express-phytotest for choosing conditions and following process of soil remediation	Petri dish	-	
461	2022	Bona	Hydrochar and hydrochar co-compost from OFMSW digestate for soil application: 2. agro-environmental properties	Petri dish	digital gauge	
462	2023	Ayoub	Evaluation of the phytotoxicity of a pesticide (TRACTOR 10E) based on Alpha-cypermethrin in	boxes	N/A	AFNORX31-201 standard

			two plant species: lentils (*Lens culinaris*) and watercress (*Lepidium sativum*)			
463	2023	Dehmani	Detoxification of olive mill wastewater by adsorption on activated clay	N/A	N/A	Dehmani *et al.,* 2020 heliyon.2020.e03164
464	2023	El Hayany	Chlorophyll performances as an indicator of compost quality: Effectiveness of liquid humic substances and compost tea	Petri dish	N/A	El Fels *et al.* 2014; Zucconi *et al.* 1981
465	2023	Elaaraj	Elaboration and structural study of Ni(II), Cu(II), Zn(II) and Co(II) complexes based on the ligand (N1Z,N2Z)-N1,N2-bis ((1H-pyrrol-2-yl) methylene) ethane-1,2-diamine with evaluation of antioxidant/antibacterial activities and cytotoxicity	Petri dish	N/A	
466	2023	Gebreyohannes	Allelopathic potential of *Lantana camara* L. leaf extracts and soils invaded by it on the growth performance of *Lepidium sativum* L.	Petri dish	N/A	
467	2023	Heivachi	Effect of the *Lactuca serriola* L extract on the cytogenetic behaviors of *Crocus sativus* L roots and its allelopathic potential	Petri dish	N/A	
468	2023	Khammassi	Phytochemical screening of essential oils and methanol extract constituents of wild *Foeniculum vulgare* mill: a potential natural source for bioactive molecules	Petri dish	N/A	
469	2023	Laskawiec	Quality assessment of sludge from filter backwash water in swimming pool facilities	plate	N/A	Phytotoxkit®; Phytotoxkit, 2004

470	2023	Lun	Isolation and identification of plant-growth inhibitory constituents from *Polygonum chinense* linn and evaluation of their bioherbicidal potential	Petri dish	ruler	
471	2023	Luo	Using time-to-event model in seed germination test to evaluate maturity during cow dung composting	Petri dish	N/A	
472	2023	Mingo	Dose-dependent positive-to-negative shift of litter effects on seedling growth: a modelling study on 35 plant litter types	pots	-	
473	2023	Motrescu	Germination and growth improvement of some micro-greens under the influence of reactive species produced in a non-thermal plasma (NTP)	containers	N/A	
474	2023	Mozejko	Effect of keratin hydrolysates obtained from feather decomposition by *Trichophyton ajelloi* on plant germination, growth and biological activity of selected arable soils under model conditions	Petri dish	N/A	Czop *et al.,* 2016
475	2023	Outman	Protein synthesis by the plant rootlet as a target for the rapid screening of anticancer drugs: the experimental model utilization of the germination of *Lepidium sativum* seeds	Petri dish	N/A	
476	2023	Photiou	Recovery of phosphates from anaerobic MBR effluent using columns of eggshell and seagrass residues and their final use as a fertilizer	N/A	N/A	Photiou and Vyrides, 2022
477	2023	Pietrini	Bismuth exposure affects morpho-physiological performances and the ionomic profile in garden cress (*Lepidium sativum* L.) plants	plastic pots	-	

478	2023	Rguez	Sesquiterpenes from *Pistacia lentiscus* L. as potential antibacterial, antifungal and allelopathic agents	Petri dish	N/A	Mancini *et al.*, 2009
479	2023	Sedefoglu	Green synthesized CuO nanoparticles using macrofungi extracts: Characterization, nanofertilizer and antibacterial effects	Petri dish	N/A	
480	2023	Senhaji	Cell growth inhibition, toxicity assessment, and correlation between chemical composition of aqueous and organic extracts of *Ajuga iva subsp pseudoiva* (DC) bric and their biological activities	Petri dish	N/A	
481	2023	Shin	Highly selective recovery of phosphate ions using a novel carbonaceous adsorbent synthesized via co-pyrolysis of spent coffee grounds and steel slags: A potential phosphatic fertilizer	Petri dish	N/A	Barderna *et al.,* 2015; Santos 2019
482	2023	Silva	Unravelling relationships between *in vivo* effects on plants and detected pesticide mixtures in freshwaters of a South-European Agro-Ecosystem	plates	with an image analysis program	Phytotoxkit, MicroBioTests, https://www.microbiotests.com/toxkit/ phytotoxicity-test-with-phytotoxkit-liquid-samples/; ISO Standard 18763
483	2023	Sourková	Establishing impact of the long-term action of waste dumps with the occurrence of waste tires on the soil environment	test board	software "Image Tool 3.0"	Phytotoxkit™ ; Phytotoxkit 2004; https://www.microbiotests.com/wp-content/uploads/2019/05/Phytotoxicity-test_Phytotoxkit-

						solidsamples_Standard-Operating-Procedure.pdf
484	2023	Topkaya	Treatment of endocrine disrupting chemicals from organized industrial zone wastewater treatment plant effluents by ozone-based AOPS: cost and toxicity assessments	Petri dish	N/A	
485	2023	Veryer	Chemical constituents of *Dysphania botrys* (L.) Mosyakin & clemants essential oil: herbicidal and antimicrobial activities	Petri dish	ruler	
486	2023	Alias	Evaluation of toxicity and genotoxicity of concrete cast with steel slags using higher terrestrial plants	Petri dish	N/A	
487	2023	Alias	Ecotoxicity evaluation of industrial waste and construction materials: comparison between leachates from granular steel slags and steel slags-containing concrete through a plant-based approach	dish	N/A	
488	2023	Ayed	Chemical composition of essential oils from eight Tunisian *Eucalyptus* species and their antifungal and herbicidal activities	Petri dish	N/A	
489	2023	Gouveia	Antineoplastic drugs in urban wastewater: Occurrence, nanofiltration treatment and toxicity screening	Test plate	software "ImageJ"	
490	2023	Jędruchniewicz	Extractability and phytotoxicity of heavy metals and essential elements from plastics in soil solutions and root exudates	Phytotoxkit	software "Image Tool 3.0"	

491	2023	Judžentiene	Allelopathic activity of Canadian goldenrod (*Solidago canadensis* L.) extracts on seed germination and growth of lettuce (*Lactuca sativa* L.) and garden pepper cress (*Lepidium sativum* L.)	Petri dish	caliper (Mitutoyo, Aurora, IL, USA)	
492	2023	Kazlauskas	Effect of graphene oxide on the uptake, translocation and toxicity of metal mixture to *Lepidium sativum* L. plants: Mitigation of metal phytotoxicity due to nanosorption	Petri dish	N/A	
493	2023	Khammassi	Investigation on chemical composition, antioxidant, antifungal and herbicidal activities of volatile constituents from *Deverra tortuosa* (Desf.)	Petri dish	N/A	
494	2023	Liwarska-Bizukojc	Effect of innovative bio-based plastics on early growth of higher plants	Test plate	image analysis using the NIS ELEMENTS AR software (Nikon, Japan)	
495	2023	Mastroberardin o	Toxicity evaluation of the contaminated area of crotone from biological indicators: a multispecies approach	Petri dish	ruler	
496	2023	Pane	Phytochemical extracts of *Dittrichia viscosa* (L.) greuter from agroecological systems: seed antigerminative properties and effectiveness in counteracting alternaria leaf spot disease on baby-leaf spinach	Petri dish	precision digital caliper (Digimatic caliper 500, Mitutoyo Corporation, Kanagawa, Japan)	De Falco *et al.*, 2021
497	2023	Shin	Enhanced selectivity and recovery of phosphate and nitrate ions onto coffee ground waste biochars via	Petri dish	N/A	

			co-precipitation of Mg/Al layered double hydroxides: A potential slow-release fertilizer			
498	2023	Siatecka	The effect of biotransformation of sewage sludge- and willow-derived biochars by horseradish peroxidase on total and freely dissolved polycyclic aromatic hydrocarbon content	N/A	software "Image Tool 3.0"	Phytotoxkit F Test; Phytotoxkit, 2004; ISO guideline 18763 (ISO, 2016)
499	2023	Thomas	Removal of zinc from concentrated galvanic wastewater by sodium trithiocarbonate: process optimization and toxicity assessment	Petri dish	N/A	
500	2023	Zhou	Colletotriauxins A−D, new plant growth inhibitors from the phytopathogenic fungus C*olletotrichum gloeosporioides*	Petri dish	visually observed and documented	
501	2023	Hkiri	Simultaneous heavy metal-polycyclic aromatic hydrocarbon removal by native Tunisian fungal species	Petri dish	N/A	Zucconi *et al*., 1981
502	2023	Kato-Noguchi	Allelopathic substances of *Osmanthus spp*. for developing sustainable agriculture	Petri dish	ruler	
503	2023	Kawa	Nanosatellite payload for research on seed germination in a 3D printed micropot	3D-printed micropot	-	
504	2023	Osipenko	Investigating the Metabolism of Plants Germinated in Heavy Water, D_2O, and $H_2{}^{18}O$-Enriched Media Using High-Resolution Mass Spectrometry	Glass vials	-	
505	2024	Abd El-Sattar	Physiological and genetical responses of L*epidium sativum* L. seeds to ultrasonic pretreatment under heat stress	Petri dish	N/A	

506	2024	Bianco	Microbial community assembly and chemical dynamics of raw brewers' spent grain during inoculated and spontaneous solid-state fermentation	wrapped in filter paper	N/A	
507	2024	Buh	Effective microorganisms technology applied to sewage sludge and tested in short exposure on *Lepidium sativum*	test plate	precision caliper	Phytotoxkit®
508	2024	Caser	The application of micro- and nano-sized zinc oxide particles differently triggers seed germination in *Ocimum basilicum* L, *Lactuca sativa* L, and *Lepidium sativum* L. under controlled conditions	Petri dish	N/A	Based on the International Rules for Seed Testing Association (2014)
509	2024	Chernysheva	Accumulation of proline, flavonoids, and organic acids in cress leaves under conditions of salt-alkaline stress	container	N/A	
510	2024	Cichy	Closing the loop: can anaerobic digestates from food waste be universal source of nutrients for plant growth?	test plate	N/A	Phytotoxkit™ test (ISO 18763:2016); Phytotoxkit, 2019; https://www.microbiotests.com/toxkit/phytotoxicitytest-with-phytotoxkit-liquid-samples/
511	2024	de Los Reyes	Age-dependent efficacy of putative dead-end trap crops *Barbarea verna* and *Lepidium sativum* on diamondback moth, *Plutella xylostella*	pot	-	
512	2024	El Finou	Phytotoxicity, antioxidant activity and chemical profile of aqueous extracts from Moroccan caper (*Capparis spinosa* L.)	Petri dish	N/A	

513	2024	Iannilli	Lithium toxicity in *Lepidium sativum* L. seedlings: exploring Li accumulation's impact on germination, root growth, and DNA integrity	Petri plate	software "ImageJ"	
514	2024	Judzentiene	Phytochemistry and allelopathic effects of T*anacetum vulgare* L (tansy) extracts on *Lepidium sativum* L. (garden pepper cress) and *Lactuca sativa* L. (lettuce)	Petri dish	caliper	
515	2024	Karadeniz-Pekgöz	A comparative evaluation of potential bioactive properties and phenolic profiles of five mediterranean Asteraceae species	Petri dish	ruler	
516	2024	Lakhloufi	Ecotoxicological evaluation of the effectiveness of natural adsorbents in the infiltration-percolation treatment of leachate	Petri dish	N/A	Helfrich *et al.,* 1999
517	2024	Lisina	The ratio of red to far-red light affects growth	pot	N/A	
518	2024	Nasircilar	Salt and heavy metal stress responses and metal uptake potentials of some leafy vegetables	pot	digital caliper	
519	2024	Vilovic	Observation of significant photosynthesis in garden cress and cyanobacteria under simulated illumination from a K dwarf star	Petri dish	-	
520	2024	Vitti	Biostimulation of humic acids on *Lepidium sativum* L	Petri dish	digital precision caliper	
521	2024	Bacmaga	Response of soil microbiota, enzymes, and plants to the fungicide azoxystrobin	plastic plates	N/A	
522	2024	Carraturo	Ecotoxicological assessment of waste-derived organic fertilizers and long-term monitoring of	Petri dish	N/A	

			fertilized soils using a multi-matrix and multi-species approach		
523	2024	Kochti	Phytochemical study on the essential oils of *Callitris glaucophylla* Joy Thomps. & L.A.S. Johnson, and assessment of their antioxidant, anti-enzymatic and allelopathic effects	Petri dish	N/A
524	2024	Labella	Germination behavior and geographical information system-based phenotyping of root hairs to evaluate the effects of different sources of black soldier fly (*Hermetia illucens*) larval frass on herbaceous crops	Petri dish	Image analysis was performed using ArcGIS version 10.8 (ESRI, Redlands, CA, USA) software
525	2024	Mendes	Spent coffee grounds as a suitable alternative to standard soil in ecotoxicological tests	Petri dish	N/A
526	2024	Moh	Allelopathy and identification of five allelochemicals in the leaves of the aromatic medicinal tree *Aegle marmelos* (L.) Correa	Petri dish	ruler
527	2024	Parmaki	Ecotoxicological assessment of biomass-derived furan platform chemicals using aquatic and terrestrial bioassays	Petri dish	N/A
528	2024	Remelli	Soil arthropods in bioindication and ecotoxicological approach: The case of the extreme environment Mefite (Ansanto Valley, Southern Italy)	Petri dish	N/A
529	2024	Shahraki	Beneficial role of coronatine on the morphological and physiological responses of cress plants (*Lepidium sativum*) exposed to silver nanoparticle	pots	ruler

530	2024	Bożym	*In vitro* chronic phytotoxicity of heavy metals and metalloids to *Lepidium sativum* (garden cress)	Petri dish	N/A
531	2024	Detzlhofer	Uptake, translocation, and metabolization of amitriptyline, lidocaine, orphenadrine, and tramadol by cress and pea	Petri dish	-
532	2024	Ranucci	The seed germination test as a valuable tool for the short-term phytotoxicity screening of water-soluble polyamidoamines	Petri dish	Dino-Lite Edge digital microscope AM7115MZT model with 5 MP resolution (VWR International s.r.l., Milano, Italy) equipped with DinoXcope software (1.5.47)
533	2024	Salehi	The exogenous application of naringenin and rosmarinic acid modulates functional traits in *Lepidium sativum*	grown in solution	-
