# Supplementary material for: A versatile Lepidium sativum bioassay for use in ecotoxicological studies
Source: Sci Rep. 2025 Sep 23;15:32653. doi: 10.1038/s41598-025-17215-7 (PMC12457589; doi:10.1038/s41598-025-17215-7)
Supplement: Supplementary file 11 — Supplementary Material 7 [file 41598_2025_17215_MOESM11_ESM.pdf]

Supplementary Figure S7:

Scans of cress seedlings treated with ecotoxic substances

Journal "Scientific Reports"

A versatile *Lepidium sativum* bioassay for use in ecotoxicological studies

Viola Maria Schulz, Claudia Scherr, Stephan Baumgartner and Alexander Tournier

Address correspondence to: Viola Schulz, MSc, Institute of Integrative Medicine,

University of Witten/Herdecke, Gerhard-Kienle-Weg 4, 58313 Witten, Germany.

E-mail: Viola.Schulz@uni-wh.de

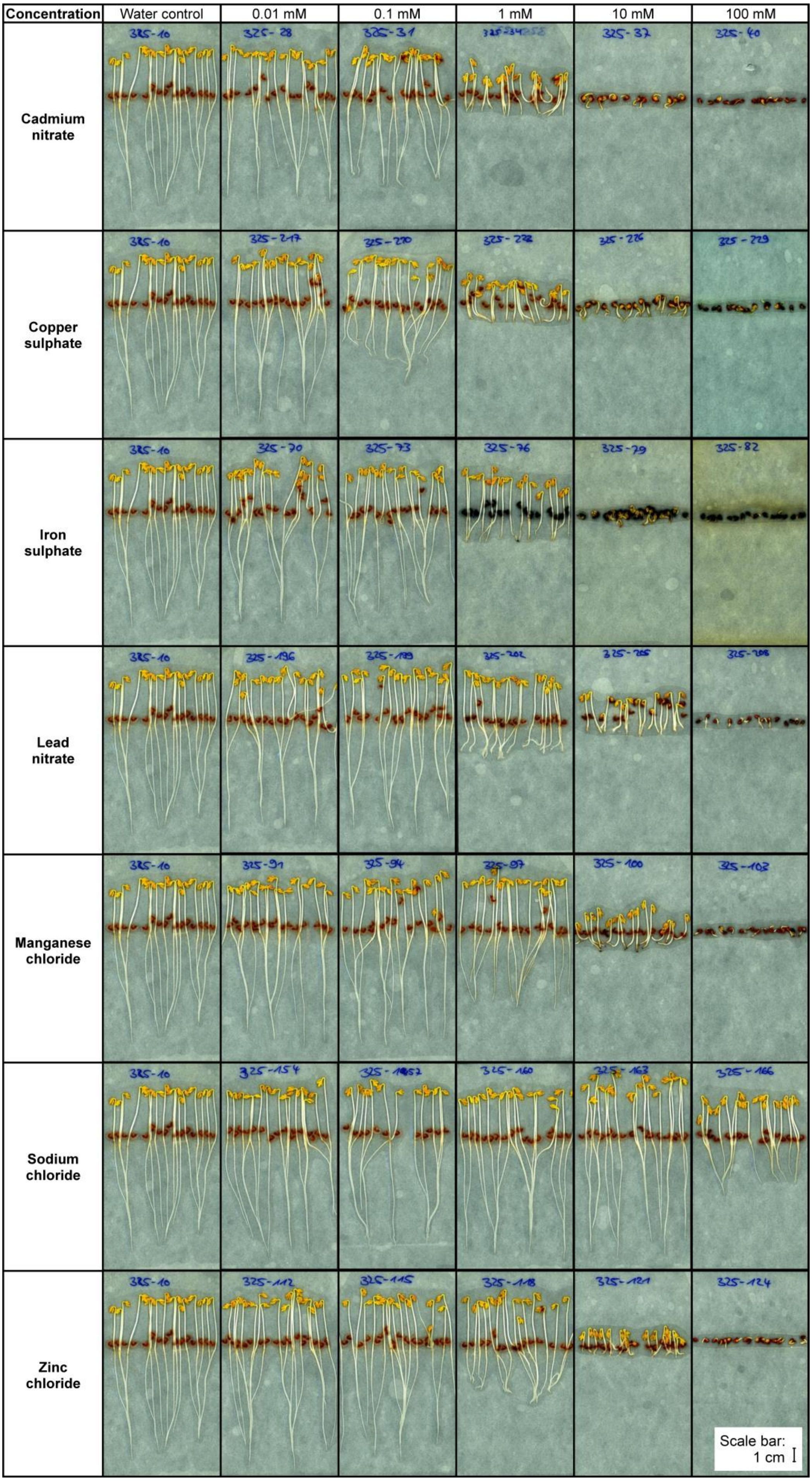
Concentration
Water control
0.01 mM
0.1 mM
1 mM
10 mM
100 mM
Cadmium nitrate
Copper sulphate
Iron sulphate
Lead nitrate
Manganese chloride
Sodium chloride
Zinc chloride
Scale bar: 1 cm
